# Metformin-restricted motility of an NRF2-activated lung cancer cell line involves NAD+ depletion, rather than AMPK- or BACH1 signaling

**DOI:** 10.1038/s41598-026-61444-3

**Published:** 2026-07-07

**Authors:** Shara Natalia Sosa Cabrera, Sophie Huber, Barbara Braunböck-Müller, Elke H. Heiss

**Affiliations:** 1 Division of Pharmacognosy, Department of Pharmaceutical Sciences, University of Vienna, Vienna, Austria; 2 Vienna Doctoral School of Pharmaceutical, Nutritional and Sport Sciences (VDS PhaNuSpo), University of Vienna, Vienna, Austria

**Keywords:** NRF2, AMPK, metformin, A549, motility, NAD+, Cancer, Cell biology, Diseases, Molecular biology

## Abstract

NRF2 is a redox-sensitive and cytoprotective transcription factor. Cancer cells exploit mutations in the NRF2-inhibitor KEAP1 and subsequent NRF2 overactivation for increased therapy resistance and malignancy. AMP-activated protein kinase (AMPK) leads to NRF2 phosphorylation and enhanced βTrCP-mediated degradation in KEAP1-deficient contexts. This study examined whether pharmacological activation of AMPK by metformin decreased levels of NRF2 and stress resilience in KEAP1-deficient A549 lung adenocarcinoma cells. Metformin treatment did not alter NRF2 half-life, expression of selected canonical NRF2 target genes, or sensitivity to cisplatin or taxol. Instead, metformin reduced abundance of BACH1, a transcription factor not only competing with NRF2 but also favoring migration of cancer cells. Testing for a potential AMPK-BACH1 motility axis in A549 cells, we observed reduced cell migration in wound closure and transwell assays upon treatment with metformin. However, depletion or overexpression of BACH1, or AMPK knockdown negated that metformin impaired cell migration primarily via BACH1 or AMPK. Metformin, as mitochondrial complex I inhibitor, increased the cellular NADH/NAD+ ratio. Supplementation with nicotinamide mononucleotide fueling NAD+ synthesis restored motility in metformin-treated cells. Thus, metformin administration does not seem to alleviate NRF2 activity but suppresses migration in KEAP1-deficient A549 cells. The latter is linked to NAD+ depletion, rather than AMPK activation or BACH1 downregulation.

**Supplementary Information:**

The online version contains supplementary material available at https://doi.org/10.1038/s41598-026-61444-3.

## Introduction

NRF2 (Nuclear factor erythroid 2-related factor 2) is a basic leucin zipper transcription factor that protects cells against oxidative, electrophilic, metabolic and proteotoxic stress. Under basal conditions it is continuously degraded through interaction with its repressor KEAP1 (Kelch-like ECH-associated protein 1) leading to NRF2 ubiquitination and degradation. Upon exposure to stressors impinging on cysteine residues of KEAP1, the suppression by KEAP1 ceases, and NRF2 becomes stabilized. Besides the main limiter valve KEAP1, also βTRCPs (β-transducin-repeat containing proteins) can tag NRF2 with ubiquitin for proteasomal degradation and adapt NRF2 activity mainly to metabolic cues. Once no longer doomed for degradation, NRF2 translocates to the nucleus, where it heterodimerizes with small MAF (musculo aponeurotic fibrosarcoma) family members and induces antioxidant and detoxifying genes through binding to ARE (antioxidant response elements) in their promoters or enhancers. The target genes of NRF2 include genes involved in redox homeostasis (e.g. *GCLC*), detoxification (e.g. *NQO1*), lipid and glucose metabolism (e.g. *G6PDH*), proteostasis (e.g. *PSMA3*) or heme/iron metabolism (*HMOX1*) (reviewed e.g. in^1,2^. BACH1, another basic leucin zipper transcription factor, opposes NRF2 by competing with NRF2 for MAF and ARE sites and usually represses ARE-mediated gene expression^3^. BACH1 also possesses unique targets that are not co-regulated by NRF2. In this context, high BACH1 levels emerged as important promoter of cancer cell invasion (in vitro) and metastasis (in vivo)^4,5^.

AMP-activated protein kinase (AMPK) is a trimeric serine/threonine kinase that acts as a central energy sensor that maintains cellular energy balance^6^. When the AMP/ATP ratio increases, AMPK is activated, promoting catabolic pathways to generate ATP while inhibiting anabolic processes that consume energy. Metformin, a widely prescribed drug for type 2 diabetes, is a well-recognized pharmacological activator of AMPK signalling, involving inhibition of mitochondrial respiratory-chain complex I, elevated AMP/ATP ratio and AMPK activity^7,8^.

In our previous studies, we identified AMPK-dependent phosphorylation sites at S374, S408, and S433 of NRF2^9^ and showed that these phosphorylation sites favour the βTRCP2-driven degradation of NRF2 in a KEAP1-depleted background^10^. Mutating the three serine residues to alanine disrupted the interaction of NRF2 with βTRCP2, thereby preventing its ubiquitination and degradation. These findings complemented the pleiotropic interactions between NRF2 and AMPK signalling (reviewed in^11^ and suggested that activated AMPK might be able to reduce cellular stress resilience by destabilizing NRF2 in cells with dysfunctional KEAP1—an approach potentially valuable in conditions characterized by KEAP deficiency and overactive NRF2 signalling^12^. Indeed, in several cancers, including non-small cell lung cancer (NSCLC), NRF2 is persistently activated by mutations in *KEAP1*, granting tumor cells an enhanced antioxidant and detoxifying capacity and survival, even under chemo- or radiotherapy^13^. Thus, we hypothesized that pharmacological activation of AMPK can blunt the protective shield of NRF2-hyperactivation. In this study we examined the influence of metformin on the KEAP1-deficient lung cancer cell line A549, which notably also lacks functional LKB1 (liver kinase B1), a known tumor suppressor and upstream activating kinase of AMPK^14^.

## Results

### Metformin does not reduce A549 biomass or cell integrity in concentrations up to 20 mM and activates AMPK

**Fig. 1:**
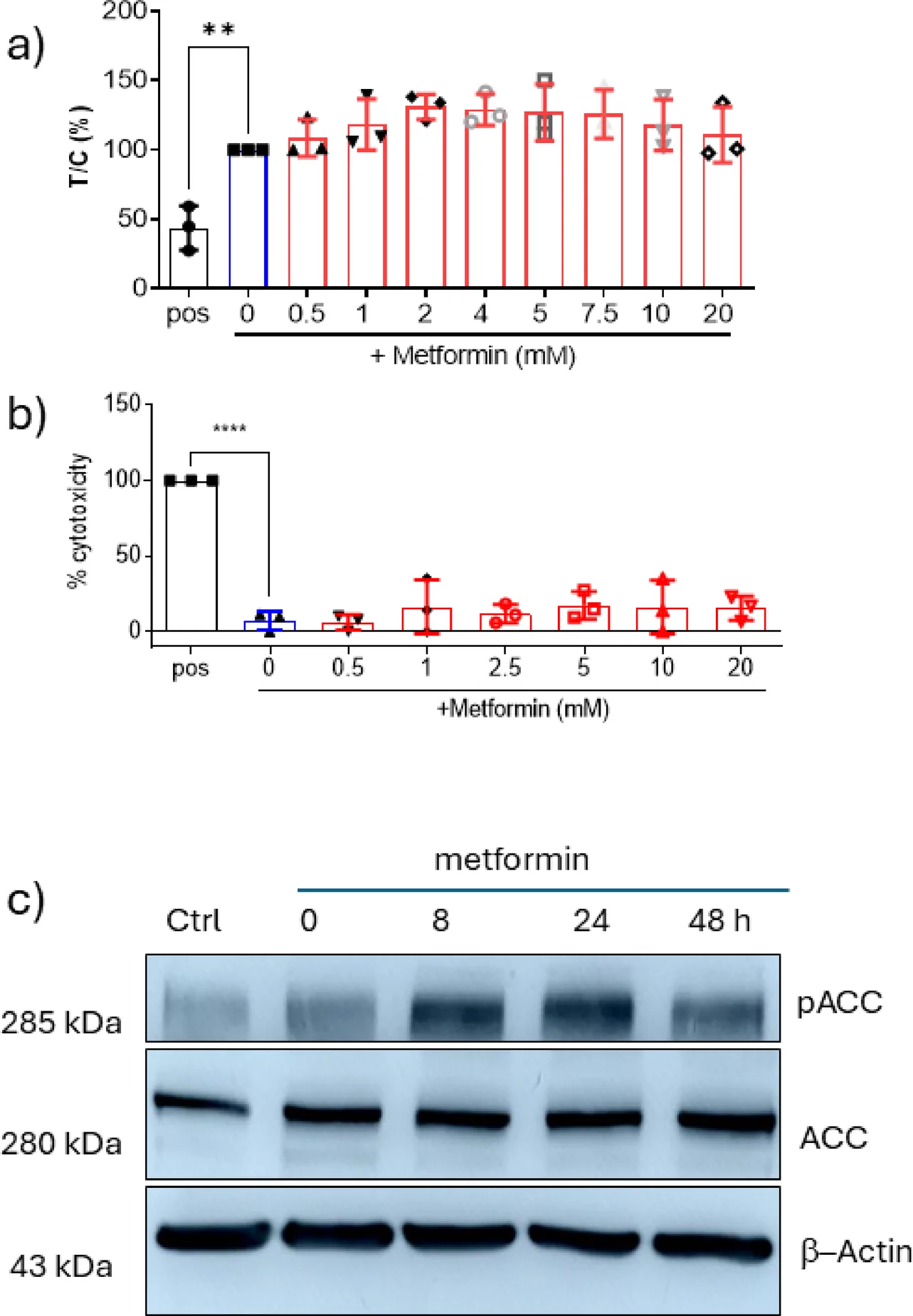
Influence of metformin on cell mass, cell integrity and AMPK activity. (**a**) A549 cells were treated for 24 h with different concentrations of metformin (as indicated) and then stained with crystal violet. After solubilizing the dye, the absorbance was measured at 595 nm. Triton X-100 (1%) was used as positive control. Absorbance values of treated cells (T) were referred to the untreated medium control (C), which was set to 100%. All other treatment groups are presented as a percentage relative to this control (T/C). (*n* = 3, mean ± SD, Kruskal Wallis/Dunn Bonferroni, **p < 0.01) (**b**) A549 cells were treated with increasing concentrations of metformin (0.5–20 mM) for 24 h, followed by measurement of membrane permeabilization using an LDH assay as described in the “Methods” section. Triton X-100 (1%) served as a positive control and the obtained value was defined as 100% cytotoxicity. Luminescence was measured using a Tecan Spark spectrophotometer (Tecan, Männedorf, Switzerland). Cytotoxicity values are expressed as a percentage relative to the Triton X-100 control. Data are presented as mean ± SD (*n* = 3). Statistical significance was assessed by Kruskal Wallis/Dunn Bonferroni tests (**p < 0.01). (**c**) A549 cells were treated with 10 mM metformin for different periods of time and probed for the levels of pACC, a downstream target of AMPK, and ACC, using western blot. β-actin was used as a loading control. Representative blots are depicted. Their uncropped versions can be found in the supplementary information.

As a first step, different concentrations of metformin (from 0.5 mM to 20 mM) were tested for their influence on A549 biomass using the crystal violet assay (Fig. 1a). Metformin did not induce any reduction after a 24 h incubation. A complementary determination of cell disintegration based on LDH release confirmed non-cytotoxicity of metformin at the used concentrations (Fig. 1b). Triton X was used as a positive control and led to a significant reduction of adherent cell mass and cell integrity. For further experiments we used either 5 or 10 mM of metformin to reach AMPK activation without compromised cell health. Successful activation of AMPK by metformin was evident by a markedly increased phosphorylation of acetyl-CoA- carboxylase (ACC) at serine 79, a known downstream target of AMPK^15^, at different timepoints as seen in Fig. 1c.

### Metformin treatment does not alter NRF2 half-life, expression of selected target genes, or the sensitivity to cisplatin or taxol in A549 cells

**Fig. 2:**
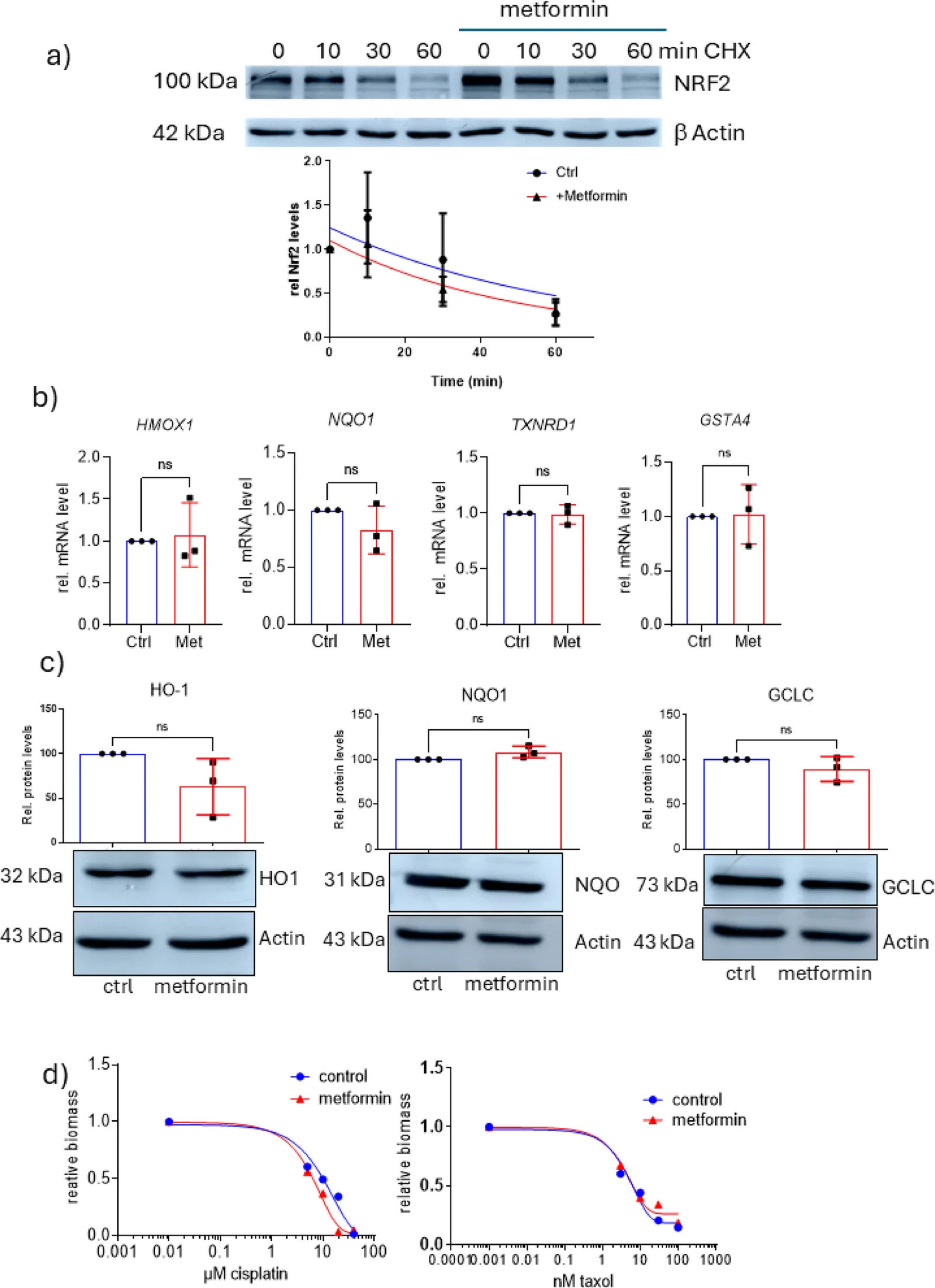
Influence of metformin on NRF2 half-life, activity of cisplatin and mRNA/protein levels of NRF2 regulated gene products. (**a**) A549 cells were treated with 10 mM metformin and 100 µM cycloheximide as explained in the method section. Samples were taken at different time points and western blot analyses for NRF2 and actin were performed. Samples without metformin were used as a control. An exemplary blot is shown together with the compiled relative NRF2/actin levels after densitometric analysis (*n* = 3, mean ± SD). The uncropped versions of the depicted membranes can be found in the supplementary information. (**b**) mRNA levels of *HMOX1*, *NQO1*,* TXRND1* and *GSTA4* were monitored after 24 h incubation with or without 10 mM metformin treatment by RTqPCR. The graph plots the relative mRNA amount compared to the untreated control. (*n* = 3, mean ± SD). (**c**) Protein levels of HO-1, GCLC and NQO1 were monitored in A549 cells after a 24 h incubation in the presence or absence of metformin by immunoblot. Actin served as loading control. Representative blots are shown (uncropped versions of the membranes in the supplementary information). The graph plots the relative amount of protein (target / leading control) compared to the untreated control sample (*n* = 3, mean ± SD) (Mann–Whitney-U-tests for **a**–**c**). (**d**) A549 cells were treated with or without metformin and different concentrations of cisplatin or taxol for 48 h. Then, a crystal violet assay was performed to measure the cisplatin or taxol -induced relative loss of biomass. Representative plots from consistent biological replicates are depicted.

Next we tested whether metformin can blunt NRF2 activity in A549 cells due to an accelerated NRF2 decay via βTrCP after AMPK-dependent phosphorylation (as indicated in^10^*)*. A half-life experiment was performed with and without treatment with metformin. As seen in Fig. 2a, the half-life of NRF2 was comparable irrespective of the presence of metformin. The same picture was obtained for A-769662, a direct allosteric activator of AMPK^16^ (Supplementary Fig. 1a) and further corroborated by unaltered NRF2 levels in A549 cells after 24 h treatment with either metformin or A-769662 (Suppl. Figure 1b). Consistently with the lacking effect on NRF2 stability and abundance, the mRNA or protein levels of selected NRF2 targets genes (*HMOX1*,* NQO1*,* GCLC*,* TXRDN1*,* GSTA4*) did not show any marked difference upon metformin treatment either (Fig. 2b, c). Lastly, the relative loss of biomass conferred by cisplatin or taxol treatment in A549 cells was comparable in control and metformin-treated cells as shown in Fig. 2d.

Overall, pharmacological activation of AMPK by metformin did not trigger an accelerated decay of NRF2 in KEAP1- depleted cells that was previously suggested from experiments with WT-NRF2 and NRF2 lacking three AMPK-dependent phosphosites.

### Metformin treatment decreases the protein and mRNA levels of BACH1

**Fig. 3:**
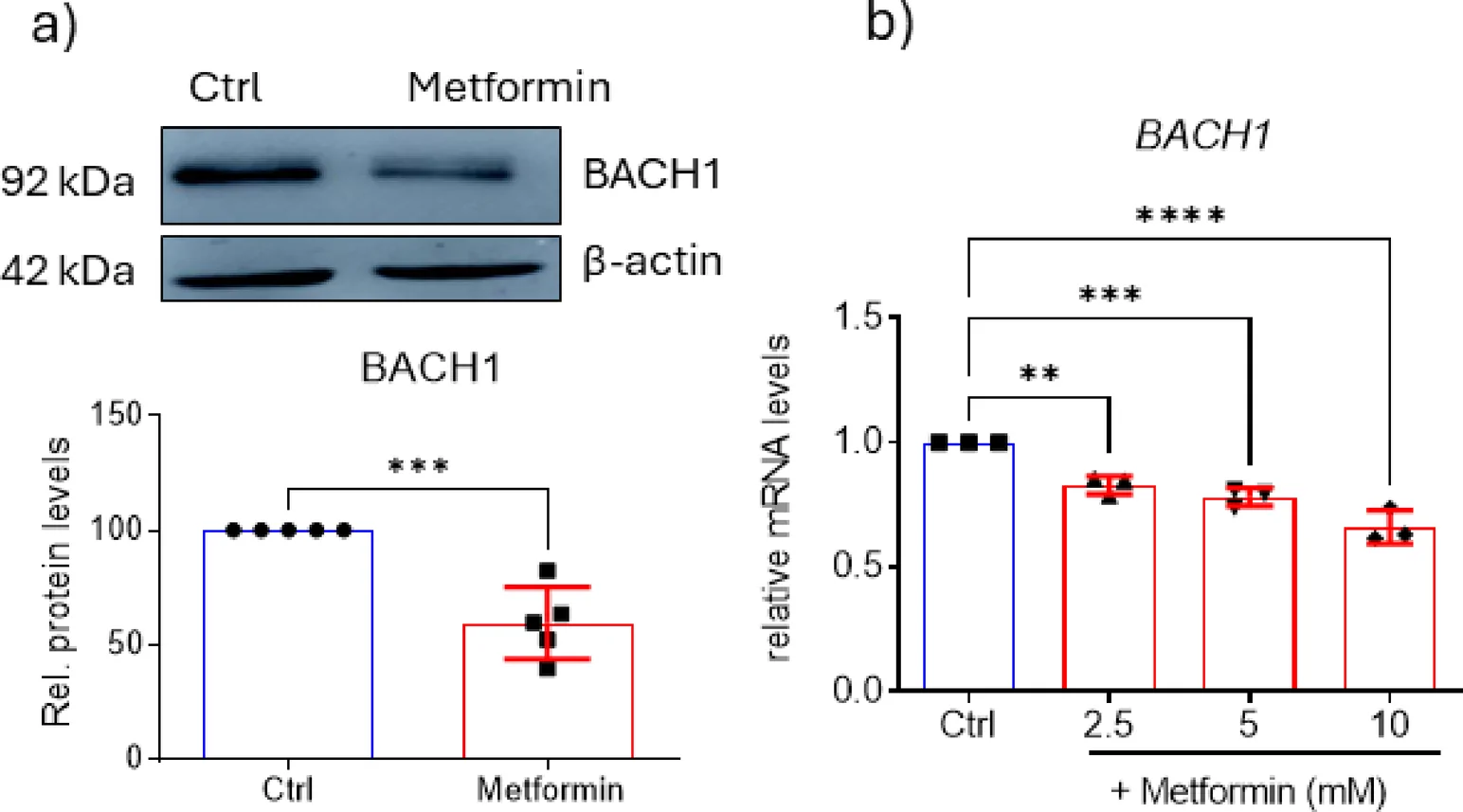
Influence of metformin on BACH1 expression. (**a**) A549 cells were treated using 10 mM Metformin for 24 h. The lysate was collected and western blot analysis for BACH1 was performed as described in the “Methods” section. β-actin was used as a loading control. Representative blots are shown with the uncropped membranes to be found in the supplement section. (**b**) For mRNA analysis, A549 cells were treated with different concentrations of metformin (2.5, 5 and 10 mM) for 24 h. RT-qPCR was performed and the relative quantity of *BACH1* mRNA is depicted in the graph. (*n* ≥ 3, mean ± SD, Mann–Whitney-U-test, *p < 0.05, **p < 0.01, ***p < 0.001, ****p < 0.0001).

Previous studies uncovered a negative regulation of BACH1 abundance by AMPK^17^. Notably, comparably high levels of BACH1, positively correlated with invasion and metastasis, can be found also in NRF2 hyperactive cancers due to high HO1 induction (and thus reduced destabilization of BACH1 by heme)^18^ and NRF2- boosted BACH1 expression as part of a negative feedback loop^19^. Based on these premises, we examined BACH1 expression and found it to be reduced on mRNA- and protein level after metformin treatment in A549 (Fig. 3).

## Treatment with metformin inhibits migration in A549 cells without involvement of BACH1 or AMPK

**Fig. 4:**
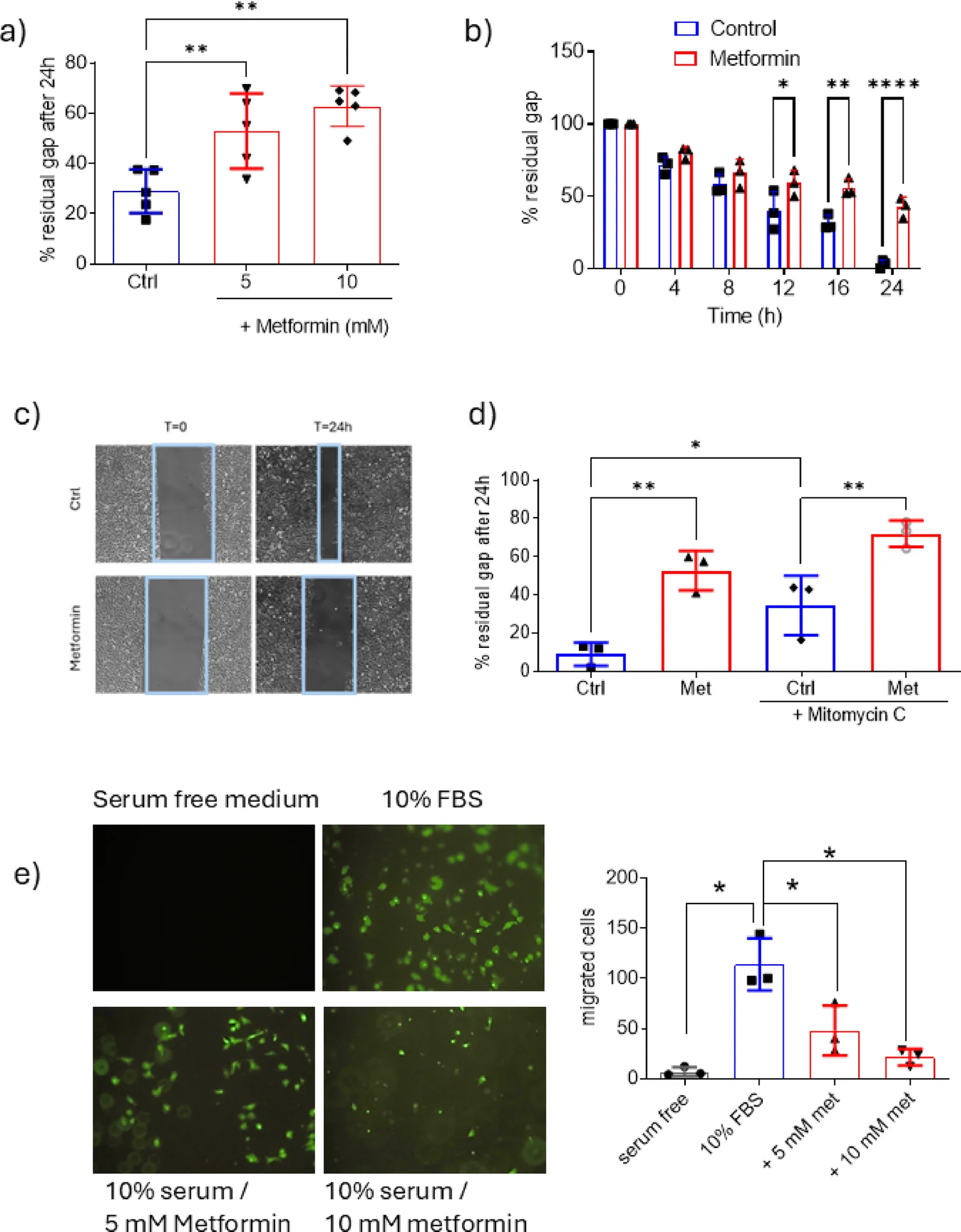
Influence of metformin on motility of A549 cells. (**a**) A549 cells were left untreated or treated with metformin (5 and 10 mM) and monitored after 24 h in their capability to close a wound inferred to the confluent monolayer (as described in the “Methods” section). The graph depicts the relative residual gaps (given as % of free area at T0 (= maximal gap)). (*n* = 5, mean ± SD, Kruskal Wallis/ Dunn/ Bonferroni *p < 0.05, **p < 0.01) (**b**) Scratch closure by A549 cells with and without treatment with metformin was monitored over time. The graph depicts the relative residual gaps after the indicated time points after the scratch. (*n* = 3, mean ± SD, *p < 0.05, **p < 0.01, ***p < 0.001, ****p < 0.0001). (**c**) The pictures show representative pictures of the 24 h time points in absence and presence of metformin. (**d**) Quantification of scratch closure in A549 cells treated with or without 10 mM metformin in the presence of mitomycin C to assess migratory capacity independent of cell proliferation. Data are presented as mean ± SD (*n* = 3). Statistical significance was determined by Kruskal–Wallis/Dunn/Bonferroni-testing (*p < 0.05, **p < 0.01, ***p < 0.001, ****p < 0.0001). (**e**) *left*: Representative images obtained from transwell migration assays performed as described in the “Methods” section. *right*: Compiled counts of migrated cells *(n* = 3; mean ± SD; *p < 0.05).

Since BACH1 positively regulates cell motility (e.g^20^. and metformin reduced BACH1 expression, a scratch assay was performed to monitor migration of A549 cells in the presence and absence of metformin. As seen in Fig. 4a), metformin treatment interferes with scratch closure at 5 mM and 10 mM monitored after 24 h incubation. A time course revealed that metformin exhibited a significant effect at 12 h of incubation. (Fig. 4b). The marked inhibition at the 24 h timepoint is illustrated in Fig. 4c). To see whether impeded scratch closure was due to inhibition of migration rather than proliferation, the assay was conducted in the presence of mitomycin C, a proliferation blocker. As shown in Fig. 4d, metformin still significantly impeded scratch closure, confirming its inhibiting effect on cell migration. Also, in a transwell assay metformin (5 and 10 mM) consistently achieved obvious inhibition of cell motility (Fig. 4e).

**Fig. 5:**
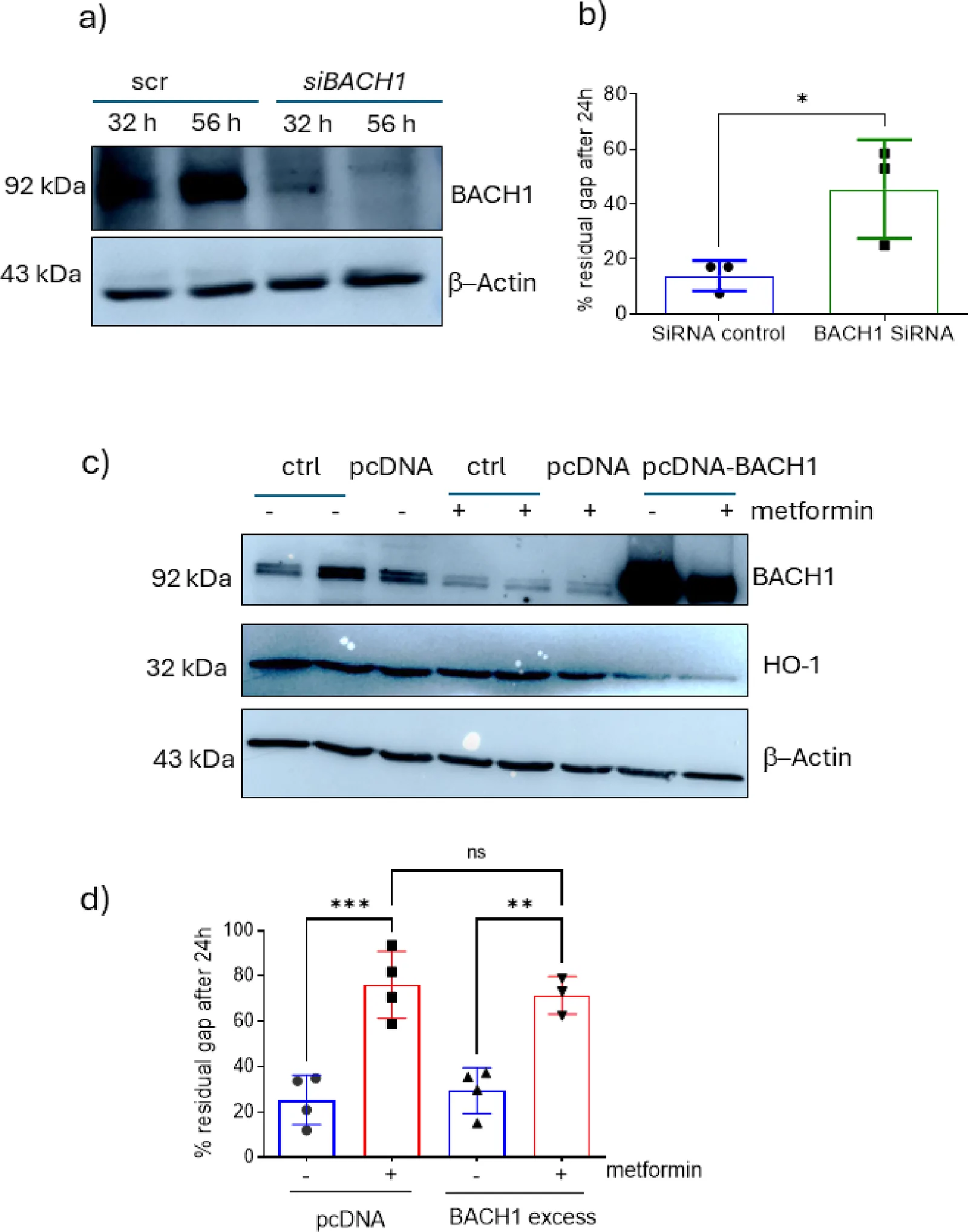
Influence of altered BACH1 levels on cell migration in A549 cells. BACH 1 was depleted in A549 cells by transfection with siRNA. (**a**) An exemplary blot shows knockdown efficiency on protein level at different time points after transfection (uncropped version in the supplement section). (**b**) The graph depicts the relative residual gap in a monolayer of control or BACH1 depleted cells at 24 h after the scratch was induced. (**c**) A549 cells were transiently transfected with empty vector (pcDNA) or an expression plasmid for BACH1 in the presence or absence of metformin. The representative blot shows the degree of obtained overexpression of BACH1 as well as the levels of HO-1, a downstream target of BACH1 repression (uncropped membranes in the supplement section). (**d**) Control and BACH1 overexpressing cells were subjected to a scratch assay in the presence and absence of 10 mM metformin for 24 h. The graph depicts the relative residual gaps. (*n* = 3, mean ± SD, Mann-Whitney-U-test (**b**) and Kruskal–Wallis/Dunn/Bonferroni (**c**) *p < 0.05, **p < 0.01, ***p < 0.001)

To examine whether metformin impaired migration via BACH1 reduction, A549 cells were either depleted of BACH1 with siRNA or transfected with an expression plasmid coding for BACH1 prior to the examination of migration (Fig. 5). Depletion of BACH1 (Fig. 5a) inhibited migration (Fig. 5b) and mimicked metformin in its anti-migratory activity in A549 cells. Cells that overexpressed BACH1 (the overexpression of BACH1 and subsequent functional repression of HO1 were confirmed by western blot (Fig. 5c)), were still inhibited by metformin in their motility to the same extent as control cells (Fig. 5d). This data suggests that BACH1 reduction can impede motility of A549 cells but it is no sufficient explanation for reduced migration in the presence of metformin.

**Fig. 6:**
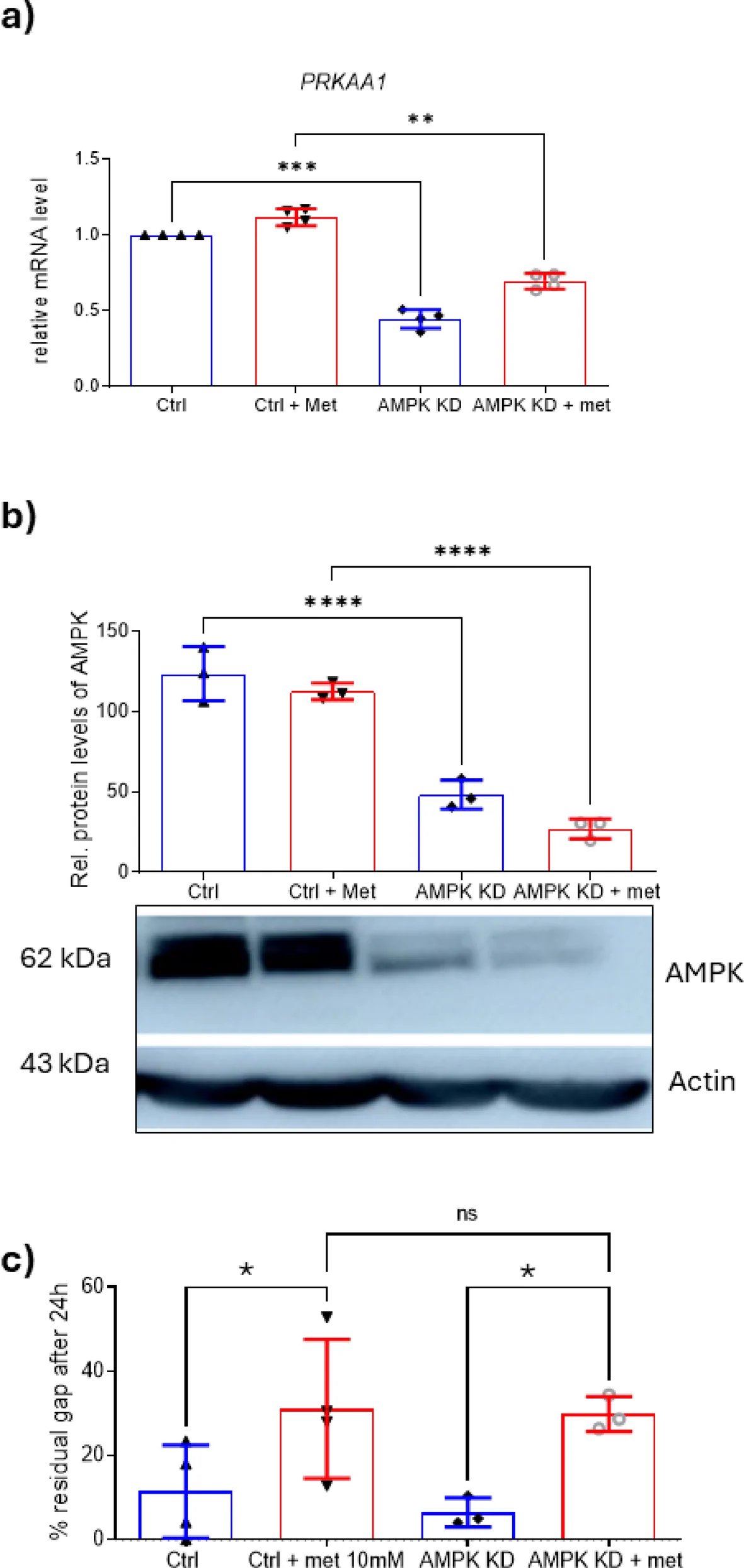
Role of AMPK for the anti-migratory effect of metformin. A549-Cas9 cells were transfected with control or gRNA targeting *PRKAA1*. Obtained knockdown efficiency was confirmed on mRNA (**a**) and protein (**b**) level. (**c**) Control and AMPKα1-depleted (KD) cells were subjected to a scratch assay in the presence and absence of metformin (10 mM). The graph depicts relative residual gaps at 24 h after the scratch was imposed. (*n* = 3, mean mean ± SD, Kruskal–Wallis/Dunn/Bonferroni *p < 0.05, **p < 0.01, ***p < 0.001, ****p < 0.0001)

To test whether the inhibition of migration by metformin is AMPK-dependent after all, AMPKα1 (the predominant catalytic subunit in A549) was depleted using the Crispr-Cas9 system. To verify a successful knockdown (KD), the protein and mRNA levels of AMPK in the obtained cell populations were checked and consistently showed ca. 50% reduction (Fig. 6a, b). Cell migration and its inhibition by metformin in these AMPK KD were not different from the mock transfected control cells (see Fig. 6c). Thus, the inhibition of motility exerted by metformin may be AMPK- independent. As 50% knockdown does not suffice to draw unambiguous conclusions, we also used a clean stable AMPK double knockout (DKO of AMPK α1/α2) clone of HEK293T cells (Suppl. Figure 2). Metformin uniformly suppressed their motility regardless of AMPK status, supporting that it inhibits cell migration independently of AMPK activation.

### Metformin treatment increases the NADH/NAD+ ratio and supplementation of NMN rescues metformin-inhibited motility in A549 cells

**Fig. 7:**
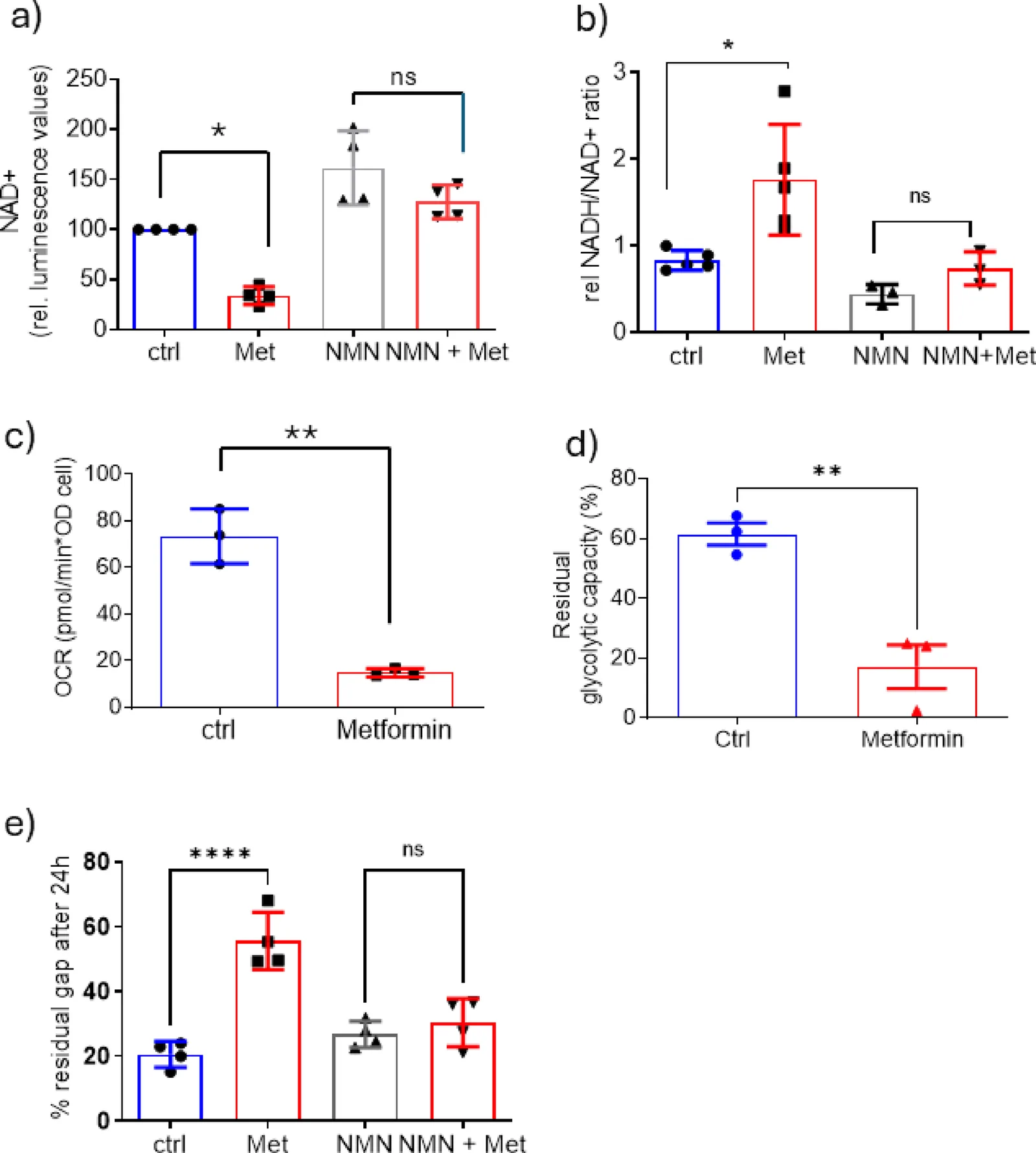
Change of cellular of NADH/NAD+ ratio by metformin and its role for metformin-inhibited migration in A549 cells (**a**) NAD+ and (**b**) ratios of NADH/NAD+ were assessed in A549 cells after 24 h treatment with or without 5 mM metformin in the absence and presence of 1 mM NMN, as described in the method section. (**c**) Basal oxygen consumption rate (OCR) and (**d**) residual glycolytic capacity in control and metformin (5 mM, 24 h)-treated A459 cells was assessed by extracellular flux analysis. (**e**) Scratch closure was monitored in A549 cells treated with metformin (5 mM, 24 h) and NMN (1 mM) as indicated. The graph depicts relative residual gaps at 24 h after the scratch was imposed. (*n* = 3, mean ± SD, Mann-Whitney-U-test, *p < 0.05, **p < 0.01, ****p < 0.0001)

Metformin is an inhibitor of the complex I of the respiratory chain^8^ and interferes with oxidation of NADH and thus the cellular NADH/NAD+ ratio^21^. Consistently, we observed reduced NAD+ signals and higher NADH/NAD+ ratios in A549 cells after 24 h metformin treatment, effects that were mitigated by supplementation of nicotinamide mononucleotide (NMN), a precursor of NAD+^22^ (Fig. 7a, b). The depletion of NAD + in metformin-treated cells was in line with a reduced oxygen consumption rate in OXPHOS and a massively reduced spare glycolytic capacity compared to control cells (as complex I inhibition renders glycolysis and lactic acid fermentation the main resort for NADH oxidation) (Fig. 7c, d). To answer whether a relative lack of NAD+ may account for the inhibited migration, a scratch assay was performed with and without supplementation of NMN. Administration of NMN could rescue the motility in the presence of metformin (see Fig. 7e). Thus, metformin seems to interfere with A549 cell migration by aggravating the reductive environment (high NADH/NAD) which is anyway high due to NRF2 hyperactivity^23^.

## Discussion

In this study, we explored the impact of metformin on NRF2 activity, BACH1 regulation, and cell motility in KEAP1/LKB1-deficient A549 lung cancer cells. Metformin could obviously not suppress global hyperactive NRF2 signalling by accelerated NRF2 degradation. However, metformin inhibited cell migration through a mechanism that occurred to be independent of BACH1 and AMPK but reliant on a relative decline in NAD+ levels.

Metformin did not reduce A549 cell biomass after 24 h at concentrations of up to 20 mM, unlike in previous reports of stronger cytotoxicity (e.g^24^. This discrepancy may be rooted in their use of the MTT viability assay, which relies on metabolic conversion of MTT (3-[5-dimethylthiazol-2-yl]-2,5-diphenyltetrazoliumbromid) to formazan and is susceptible to interference from redox/metabolism-altering compounds like metformin^25^. A performed LDH assay confirmed that metformin does not exert any cytotoxicity under the employed conditions.

Despite previous evidence that AMPK-driven phosphorylation promotes βTrCP-mediated degradation of NRF2 in murine KEAP1-deficient embryonal fibroblasts^10^, metformin did not alter NRF2 decay in human A549 cells. The ADaM (allosteric drug and metabolite)-site directed AMPK activator A-769662^16^ was similarly ineffective, and neither metformin nor A-769662 downregulated βTrCP abundance (Supplementary Fig. 1). Pharmacological AMPK activation may induce multiple diverse responses beyond NRF2 phosphorylation that mask, override or compensate for the (weak) phosphorylation-dependent impact on NRF2 stability or act in a species-or context dependent manner. Several studies reported metformin lowering NRF2 in A549 at comparable doses^26,27^, which may reflect setting-dependent variables (medium, serum, confluency, co-treatments, incubation times).

Metformin significantly lowered BACH1 protein and mRNA in A549 cells, consistent with prior reports in lens epithelial cells and with AMPK’s negative regulation of *Bach1* in murine fibroblasts^17,28^. BACH1 does not only present as repressive competitor of NRF2 but is also connected with cancer invasiveness involving factors like matrix metalloprotease (MMP) 1, hexokinase (HK)2 or glycerine aldehyde phosphate dehydrogenase (GAPDH)^5,29,30^. We observed reduced motility after metformin exposure in A549 cells, in line with reports from other cell types^31,32^. However, rescue experiments showed that BACH1 reduction (alone) cannot explain the decreased cell mobility by metformin. Moreover, metformin did not lower expression of *HK2*,* GAPDH*,* or HTRA3* (Suppl. Figure 3), genes (co)-regulated by BACH1 and involved in promotion of motility^33^ in our system. Likewise, AMPK knockdown failed to restore migration in the presence of metformin, arguing against a central role for AMPK activation. Dong et al. proposed that metformin treatment in A549 cells increases AMPK-driven expression of microRNA-7, which contributes to reduced cell motility and growth. However, their study lacked experiments in AMPK-depleted cells, leaving open the possibility that the observed effects may still occur independently of AMPK^34^.

Unlike direct AMPK activators (such as AICAR or A-769662), metformin acts indirectly by shifting the AMP/ATP ratio via inhibition of mitochondrial complex I^8^. Complex I blockade also impairs NADH oxidation to $\:{\mathrm{N}\mathrm{A}\mathrm{D}}^{+}$, elevating the $\:\mathrm{N}\mathrm{A}\mathrm{D}\mathrm{H}/{\mathrm{N}\mathrm{A}\mathrm{D}}^{+}$ratio. In A549 cells with hyperactive NRF2 and comparably low NAD+^25^, metformin further increased $\:\mathrm{N}\mathrm{A}\mathrm{D}\mathrm{H}/{\mathrm{N}\mathrm{A}\mathrm{D}}^{+}$, and NMN supplementation could restore migration. Thus, metformin seems to inhibit motility primarily via NAD+/NADH imbalance, not via AMPK-, or BACH1 signaling. Consistently, FK866, an inhibitor of the NAD+ biosynthesis enzyme nicotinamide phosphoribosyl transferase, sensitized pancreatic cancer cells to metformin^35^, and the $\:{\mathrm{N}\mathrm{A}\mathrm{D}}^{+}$pool was linked to tumor aggressiveness (e.g^36,37^. Previous experiments in our lab could exclude reactive oxygen species (ROS)/mitochondrial superoxide production or ATP depletion as primary contributors to the reduced motility in metformin-treated A549.

Taken together, metformin suppresses A549 cell motility by inducing reductive overload through complex I inhibition and an NADH/NAD+ imbalance. Although AMPK activation and BACH1 downregulation occur in our setting, they do not account for the reduced migration in the presence of metformin. Already existing observations on motility, NAD+ pool, and BACH1 in metformin-treated cells could now be extended by probing their causal links. Obtained insights underscore the importance and value of (i) metabolic and redox perturbations in shaping cancer cell behaviour and (ii) the careful look beyond *bona fide* targets of bioactive molecules.

An obvious limitation towards generalization of our findings is the in vitro, single-cell-line setup with only restricted dose/time regimens. The millimolar metformin concentrations used (although not uncommon for in vitro studies due to cell culture conditions (serum, glucose, lack of effective transport mechanisms/organ accumulation) increase the risk of unintended off-target effects. Moreover, mapping of the NAD+-motility signaling axis (i.e. questions about correlation of concentration-dependent metformin-effects on motility and NAD+/NADH ratio, mitochondrial vs. cytosolic NAD+, or primarily affected enzymes) and the importance of KEAP1 remained unresolved issues within this study. Concerning the latter, metformin failed to inhibit migration in HT1299 cells, a lung cancer cell line with functional KEAP and NRF2 control. However, knockdown of KEAP1 could not render HT1299 susceptible to metformin action, nor did NRF2 knockdown or KEAP overexpression markedly attenuate metformin’s inhibition of A549 motility, excluding a simple straight NRF2/KEAP signalling axis to NAD + and cell migration (Supplemental Fig. 4). Future studies are clearly warranted to test generalizability across different cancer cell types, KEAP1 and also LKB1/AMPK backgrounds and assess whether clinically relevant metformin doses similarly drive NAD+-dependent effects on cancer metastasis in vivo.

## Methods

### Chemicals

Metformin hydrochloride was purchased from Cayman Chemicals, dissolved in water to a 100 mM stock solution, sterile filtrated, aliquoted and stored at − 20 °C. Cycloheximide (CHX) and cisplatin were purchased from Sigma-Aldrich and dissolved under sterile conditions in 100% DMSO into 40 mM and 100 mM stocks, respectively, aliquoted and stored at − 20 °C. A769662 was purchased from MedChemExpress, dissolved in 100% DMSO for 50 mM stocks, aliquoted, and stored at − 20 °C.

### Cell culture

A549-Cas9 cells were obtained as a kind gift from Karin Ortmayr (Dep. Pharmaceutical Sciences, University of Vienna). A549 and HT1299 cells were obtained from ATCC. Cell lines were grown at 37 °C/5% CO_2_ in Dulbecco´s Modified Eagle´s Medium (Sigma-Aldrich) (DMEM) supplemented with 10% heat inactivated FBS (Biowest), 2 mM L-glutamine, 100 IU/ml penicillin, and 100 µg/ml streptomycin. To ensure optimal growth of the cells, they were passaged once they reached 90% confluency. The cells were used up to passage 35.

### Biomass stain by crystal violet

The assay was carried out in a 96-well-plate, and 1 × 10^4^ cells were seeded per well in 100 µl of medium. After 24 h the cells were treated with different concentrations of metformin. As a positive control 1% Triton X-100 (Sigma Aldrich) was used. After 24 h of treatment, medium was removed and cells were washed with PBS. Then, 75 µl of crystal violet solution (1% crystal violet, 20% methanol (v/v)) were added to each well for 5 min. Then the violet solution was discarded, residual unbound dye was washed off with water, and the plate was left to dry overnight. Once dry, the bound dye was solubilized with 150 µl of 0.1 M citrate/EtOH (1:1) and the absorbance was measured at 595 nm using the TECAN Sunrise.

### Assessment of cytotoxicity via released lactate dehydrogenase (LDH)

Cells were seeded at a density of 1.5 × 10⁴ cells per well in 96-well plates and allowed to adhere overnight under standard culture conditions (37 °C, 5% CO₂). Cells were then treated with metformin at concentrations ranging from 0.5 to 20 mM for 24 h. Untreated cells served as negative control, while cells treated with 1% Triton X-100 for 24 h were used as positive control of maximal membrane permeabilization. Following treatment, cell culture supernatants were collected, and LDH activity was measured using the Promega LDH Cytotoxicity Assay Kit according to the manufacturer’s instructions. Briefly, supernatants were incubated with an equal volume of LDH detection reagent for 1 h at room temperature, protected from light, prior to measuring luminescence using a microplate reader. Cytotoxicity was calculated as a percentage of LDH release relative to the Triton X-100-treated control.

### Protein extraction, SDS PAGE and Western blot

Following our protocol reported in^38^, total cell lysates were obtained by incubation of washed cells with NP40 lysis buffer (150 mM NaCl, 1% NP40, 10 mM DTT in 50 mM Tris (pH 8.0)) with a mixture of protease and phosphatase inhibitors on ice for 10 min. Cell lysates were then scraped from the culture plates into reaction tubes, followed by short sonication (15 s, 90% power). After centrifugation for 15 min (11,000×*g* at 4 °C), supernatants containing total soluble cell protein were collected. 25 µg of protein was separated on 7.5% or 10% sodium dodecyl sulfate-polyacrylamide gels by denaturing electrophoresis (SDS-PAGE) and transferred to a methanol-activated 0.2-mm polyvinylidene difluoride membrane (PVDF). The membranes were blocked with 5% bovine serum albumin (BSA) in TBST for 1 h at room temperature and incubated with specific primary antibodies at 4 °C overnight (antibody dilution 1:1,000 in TBST) (anti-GSC: Santa Cruz #SC166382, anti-HO-1: Enzo Life Sciences #ADI-SPA-895 F, anti-NQO1: Cell Signaling #3187s, anti-BACH1: Santa Cruz #271211, anti-AMPK: Cell Signaling #2532, anti-NRF2: Proteintech # 16396-1-AP), followed by HRP-conjugated secondary antibody incubation at room temperature for 1 h. Signal intensity was measured using enhanced chemiluminescence (ECL) and an Amersham ImageQuant 800 imager (Cytiva). The densitometric analysis of the bands was measured using proprietary Cytiva software.

### RNA extraction and RT-qPCR analyses

As previously described^38^, total RNA was extracted using an RNA isolation kit (#845- KS-2040250, IST Innuscreen GmbH) according to the manufacturer’s protocol. RNA integrity was assessed by agarose gel electrophoresis, and quantification was achieved by spectrometric measurements using a NanoDrop 2000 (Thermo Fisher Scientific), also allowing a purity check via the A260/A280 ratio (≥ 2.0). cDNA was synthesized from 1 µg of RNA using a High-Capacity cDNA Reverse Transcription Kit (Thermo Fisher- #4368813), again following the manufacturer’s instructions. cDNA quantitation was performed using the Luna Universal qPCR Master Mix (#M3003E, New England Biolabs) and the qTower Iris (Analytik Jena) using a cycling protocol of one denaturation step (10 min at 95 °C) and up to 45 amplification cycles (15 s at 95 °C, 30 s at 60 °C) as well as melting curves between 55 and 95 °C. The quality of the amplification was ensured by a single peak in the melting curve, only one amplicon of the desired size on an agarose gel, and no amplification in the negative (no template) control. Data analysis was performed by using the (2^–ΔΔCt^) method. Used gene-specific primers were designed with the help of Primer3 and BLAST (*BACH1* fw: GCCTCGCACAATATGGTTGAT rv: GCCTCGCACAATATGGTTGAT; *PRKAA* fw: AGGAAGAATCCTGTGACAAGCAC rv: CCGATCTCTGTGGAGTAGCAGT; *HMOX1* fw: CCAGGCAGAGAATGCTGAGTTC rv: AAGACTGGGCTCTCCTTGTTGC; *NQO1* fw: CCTGCCATTCTGAAAGGCTGGT rv: GTGGTGATGGAAAGCACTGCCT; *TXRDN1* fw: GTTACTTGGGCATCCCTGGTGA rv: CGCACTCCAAAGCGACATAGGA; *GSTA4*: fw: ACAGACCCGAAGCATTCTCCAC rv: AGTTCCAGCAGATCCAGTGTCC; *HK2* fw: GAGTTTGACCTGGATGTGGTTGC rv: CCTCCATGTAGCAGGCATTGCT; *GAPDH* fw: GTCTCCTCTGACTTCAACAGCG rv: ACCACCCTGTTGCTGTAGCCAA; *HTRA3* fw: CAAGAAGTCGGACATTGCCACC rv: CGTTGTCACTGTGTTCTGTAGGG); *NFE2L2* fw: CACATCCAGTCAGAAACCAGTGG rv: GGAATGTCTGCGCCAAAAGCTG; *KEAP1* fw: CAACTTCGCTGAGCAGATTGGC rv: TGATGAGGGTCACCAGTTGGCA) and ordered from Thermo Fisher Scientific. Beta *ACTIN* (fw: CACCATTGGCAATGAGCGGTTC rv: AGGTCTTTGCGGATGTCCACGT) was taken as a reference gene in the used experimental setups due to its stable expression throughout the employed treatment regimens and its Ct values close to the ones of the investigated target genes.

### Assessment of oxygen consumption rate and spare glycolytic capacity

According to previously published protocols^38^, cells were seeded and treated as desired. On the day of measurement, cells were scraped and plated (1 × 10^5^ cells/well) in XF24e-cell culture plates precoated with Corning Cell-Tak adhesive (#CLS354240, Sigma Aldrich). Cells were incubated in an XF assay medium (pH 7.4/37 °C) (#103575-100, Agilent Technologies) supplemented with 2 mM glutamine, 1 mM pyruvate, and 25 mM glucose for 1 h at 37 °C in a non-CO_2_ incubator. Real-time extracellular acidification (ECAR) and oxygen consumption (OCR) rates were measured in an XF24e Flux Analyzer (Seahorse Bioscience-Agilent Technologies). ECAR was monitored under basal conditions, as well as after rotenone (Rot, 0.5 µM)/antimycin A (AA; 0.5 µM) and 2-DOG (deoxyglucose) injections (50 mM), using the proprietary glycolytic rate assay protocol from Agilent (#103344-100). Data were analysed using the Wave software package from Agilent Technologies.

### Assessment of cell migration via wound closure/scratch assays

This cell migration assay was performed either in a 12-well plate (ca. 3 × 10^5^ cells) or in a 6-well plate (ca. 7.5 × 10^5^ cells). 24 h after seeding the medium was removed, and a scratch was made in the confluent cell monolayer using a 1 mL sterile tip. Wells were washed and filled with respective medium +/- metformin. Pictures were taken (Olympus PEN Lite E-PL7 digital mirrorless camera) directly after the scratch (T0) and then at different time points to monitor scratch closure over time. The analysis was performed using ImageJ. The cell-free wound area at time 0 (T0) was measured and defined as 100%. At subsequent time points, the remaining wound area was quantified at identical coordinates, and wound closure was calculated as the percentage cell-free area relative to T0. To assess the contribution of cell migration to scratch closure, independently of proliferation, cells were pre-treated with 5 µg/mL mitomycin C for 1 h to inhibit cell division, followed by three washes to remove residual compound. Subsequently, the assay was performed as described above.

### Assessment of cell migration by a transwell assay

A549 cells were stained by incubation with CellTracker Green CMFDA (# C7025 from Thermo Fisher) at a final concentration of 5 µM in serum-free Dulbecco’s Modified Eagle Medium for 30 min at 37 °C and 5% CO₂. Labelled cells were harvested and then seeded into the upper chamber of transwell inserts (8 μm pore size), while the lower chamber was filled with medium containing 10% fetal bovine serum as a chemoattractant. Cells were treated with metformin at final concentrations of 5 mM and 10 mM. After incubation for 24 h at 37 °C and 5% CO₂, non-migrated cells were removed from the upper surface of the membrane using a cotton swab. Inserts were gently washed with phosphate-buffered saline and maintained in PBS for imaging. Migrated cells (on the bottom side of the membrane) were visualized using a fluorescence microscope (FITC channel) and representative images per insert were acquired at 20× magnification. Quantification was done in a blinded fashion by ImageJ-assisted cell counting (average of four different fields/condition) from three biological replicates.

### Crispr-Cas9 mediated knockdown of AMPK

A549Cas9 cells were seeded in a 6-well-plate (7.5 × 10^5^ cells). When they were sub-confluent (approximately 24 h later) they were transfected using gRNA (75 pmol) targeting the *PRKAA1* gene (encoding AMPKα1) and Lipofectamine 3000 (7.5 uL) in Opti-MEM medium. To ensure that the transfection mix alone did not influence the experimental readouts, a respective mix was also prepared without the addition of gRNA. The gRNA was purchased from Invitrogen #A35533 (CRISPR1005770_SGM).

### SiRNA-mediated knockdown of BACH1, NRF2 and KEAP1

For knockdown of BACH1, 3 × 10^5^ A549 cells per well were seeded in a 12 well plate. The transfection mix (per well) consisted of 3 µL Lipofectamine and 50 µL of Opti-MEM medium (tube A) and 30 pmol of the desired siRNA and 50 µL of Opti-MEM (tube B). Solutions from tube A and B were mixed, incubated for 12 min and then added to the cells. After an incubation of 24 h, cells were subjected to motility assays, and knockdown efficiency was determined (by qPCR or immunoblot). The siRNA for *BACH1* (# HU-04988-1) as well as a scrambled control were purchased from Eupheria Biotech.

For knockdown of NRF2 or KEAP1, cells were cultured in antibiotic-free full growth medium. 3 × 10^5^ A549 cells per well were seeded in a 12 well plate. The transfection mix (per well) contained a mix of 2.5 µL of Dharmafect and 100 µL of Opti-MEM medium (tube A) and 25 pmol of the desired siRNA and 100 µL of Opti-MEM (tube B). Each tube was incubated for 5 minutes at room temperature and then combined. After another 20 min incubation, 200 µL of the mix were added to the cells. Incubation was done for ca. 24 h prior to assessment of migration and knockdown efficiency. The siRNA pools for *NFE2L2* (#L-003755-00-0005) and *KEAP* (#L-012453-00-0005) as well as the scrambled controls were purchased from Horizon Discovery.

### Overexpression of BACH1 and KEAP1

3 × 10^5^ A549 cells per well were seeded in a 12-well plate. The transfection mix (per well) consisted of 3 µL Lipofectamine and 50 µL of Opti-MEM medium in tube A and 0.5 µg of plasmid DNA plus 2 µL of P3000 in 50 µL of Opti-MEM medium (tube B). Solutions of tube A and B were mixed, incubated for 12 min and then added to the cells. The incubation was done for ca. 24 h prior to assessment of overexpression and readouts of interest. The expression plasmids for BACH1 (Plasmid #159130) and KEAP1 (Plasmid # #28023) were obtained from Addgene. As control an empty pcDNA vector was used.

### Assessment of NRF2 half-life

A549 cells were seeded in a 12 well plate (3 × 10^5^ cells) and grown for 24 h. Then cells were preincubated with or without metformin for one hour before cycloheximide (inhibitor of protein synthesis; 100 µM) was added for the indicated periods of time and the cells were harvested for western blot analysis of NRF2.

### Assessment of cellular NADH/NAD+ ratios

The protocol is adapted from^39^. Briefly, cells were plated in 6-well dishes at 8 × 10^4^ cells per well, allowed to adhere overnight, then washed twice in PBS, and cultured in 2 ml of medium with the indicated treatment for 24 h. After detachment, 7.5 × 10^4^ cells were then washed three times in ice cold PBS and lysed in 100 µL of ice cold lysis buffer (1% Dodecyltrimethylammonium bromide (DTAB) in 0.2 N NaOH diluted 1:1 with PBS), immediately flash frozen and kept at − 80 °C for 24 h. NAD+/NADH ratio was measured using a modified version of instructions provided with the NAD/NADH Glo Assay Kit (Promega). For NADH measurement, 20 µL of the freshly thawed lysate was transferred to PCR tubes and incubated at 75 °C for 30 min which allows for base-mediated degradation of NAD+. For NAD+ measurement, 20 µL of the freshly thawed lysate was transferred to PCR tubes containing 20 µL lysis buffer and 20 µL 0.4 N HCl. NAD+ samples were incubated at 60 °C for 15 min, where acidic conditions result in selective degradation of NADH. Following respective incubations, samples were allowed to equilibrate to room temperature for 8 min and then quenched with the respective neutralizing solution of 20 µL 0.25 M Tris in 0.2 N HCl (NADH) or 20 µL 0.5 M Tris base (NAD+). Following sample prep, manufacturer’s instructions were used for enzyme-linked luminescence-based NAD+ and NADH measurement.

### Statistics

Unless stated otherwise, at least three independent biological replicates were performed for the experiments. The bar graphs depict the mean ± standard deviation (SD). Groups of data (Gaussian-normality/homogenous variance not secured) were compared via Mann-Whitney-U-test, or Kruskal–Wallis and post-tests by using GraphPad Prism 9 software. The details for each figure can be found in the figure legend. Differences were considered as significant if *p* < 0.05.

## Supplementary Information

Below is the link to the electronic supplementary material.

Supplementary Material 1

## Data Availability

The data that support the findings of this study are available from the corresponding author, EHH, upon reasonable request.
