# Supplementary material for: Metformin-restricted motility of an NRF2-activated lung cancer cell line involves NAD+ depletion, rather than AMPK- or BACH1 signaling
Source: Sci Rep. 2026 Jul 7;16:31264. doi: 10.1038/s41598-026-61444-3 (PMC13642031; doi:10.1038/s41598-026-61444-3)
Supplement: Supplementary file 1 [file 41598_2026_61444_MOESM1_ESM.pdf]

Supporting Information

Metformin-restricted motility of an NRF2-activated lung cancer cell line involves NAD+ depletion, rather than AMPK- or BACH1-signaling

Shara Natalia Sosa Cabrera[1,2], Sophie Huber[1], Barbara Braunböck- Müller [1], Elke H Heiss[1].

[1]Department of Pharmaceutical Sciences, Division of Pharmacognosy, University of Vienna, Vienna, Austria

[2]Vienna Doctoral School of Pharmaceutical, Nutritional and Sport Sciences (VDS PhaNuSpo), University of Vienna, Austria

Corresponding author: elke.heiss@univie.ac.at

Supplementary Figures 1- 5

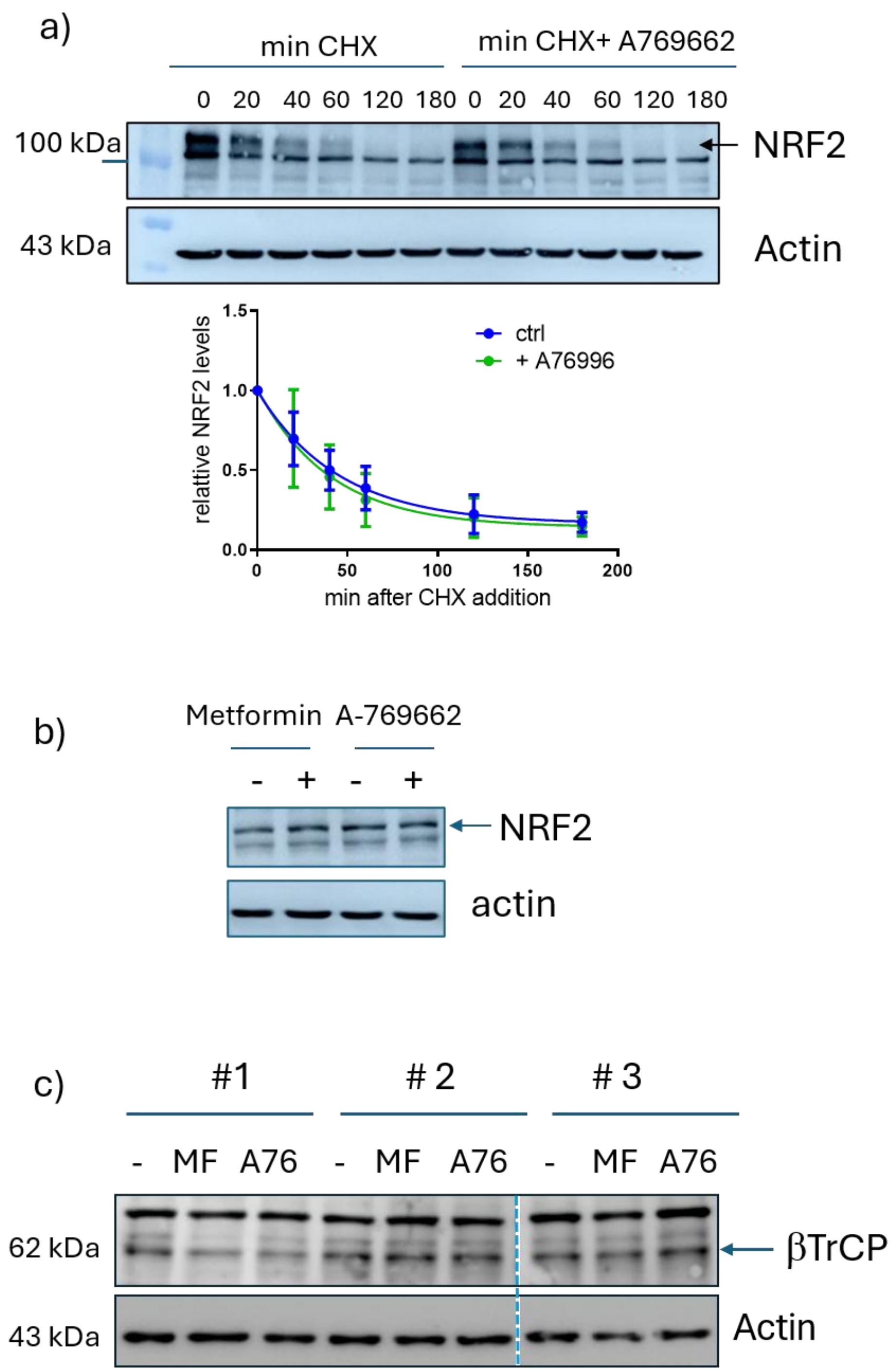

Supplementary Figure 1. AMPK activation by A-769662 does not alter NRF2 half-life or abundance of NRF2 or βTrCP in A549. (a) A549 cells were preincubated for 1h with or without the allosteric AMPK activator A-769662 (50 µM) before cycloheximide (100 µM) was added for the indicated periods of time. Total cell lysates were probed for NRF2 and actin (a representative immunoblot is shown). The graph depicts compiled relative NRF2/actin ratios of three independent experiments. b) A549 cells were treated as indicated (metformin 10 mM;

A-769662 50 µM) for 24 h prior to immunoblot analysis for NRF2 and actin. A representative blot is depicted. c) A549 cells were left treated with solvent (-), metformin (MF; 10 mM) or A769662 (A76; 50 µM) for 24 h prior to immunoblot analysis for βTrCP1/2 (antibody sc-390629 from Santa Cruz (1:200)) and actin. Blot shows results from three independent biological replicates are depicted (#1-3).

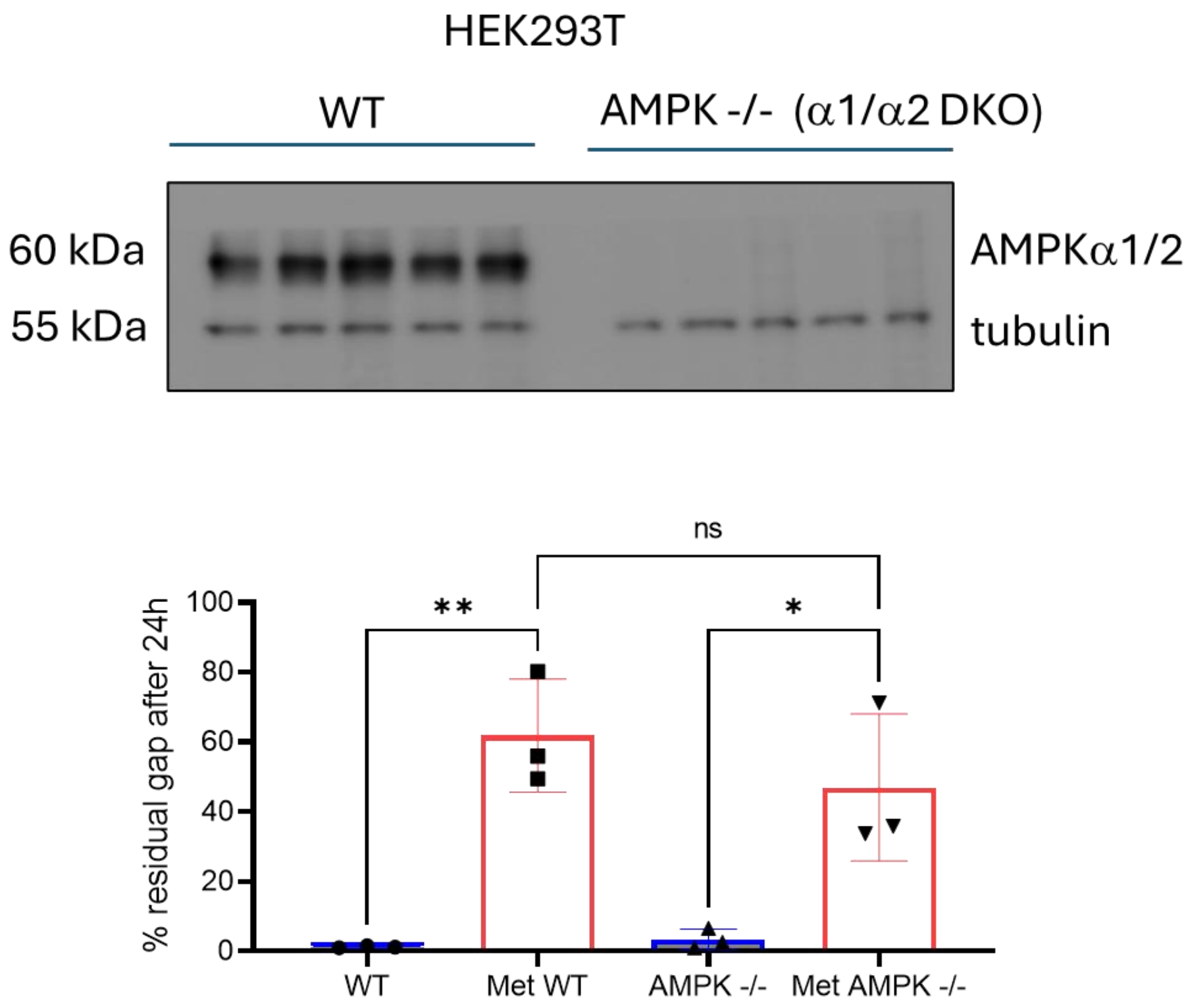

Supplementary Figure 2. Cell motility in metformin-treated AMPK α1/α2 double knockout HEK293 cells

HEK293T AMPK (α1/α2)-/- double knockout (DKO) cells were kindly provided by Prof A. Pause published in [1]. a) Immunoblot analysis of different batches of cell lysates from WT and DKO HEK293T cells were probed for AMPK α and tubulin as loading control. b) Migration of WT and AMPK -/- HEK293T cells was assessed in the presence and absence of 10 mM metformin. The gap closure was monitored for 24h and the residual gap after 24h is depicted in the graph. (n=3, mean ± SD, Mann-Whitney-U-test, $^{*}p < 0.05$, $^{**} p<0.01$)

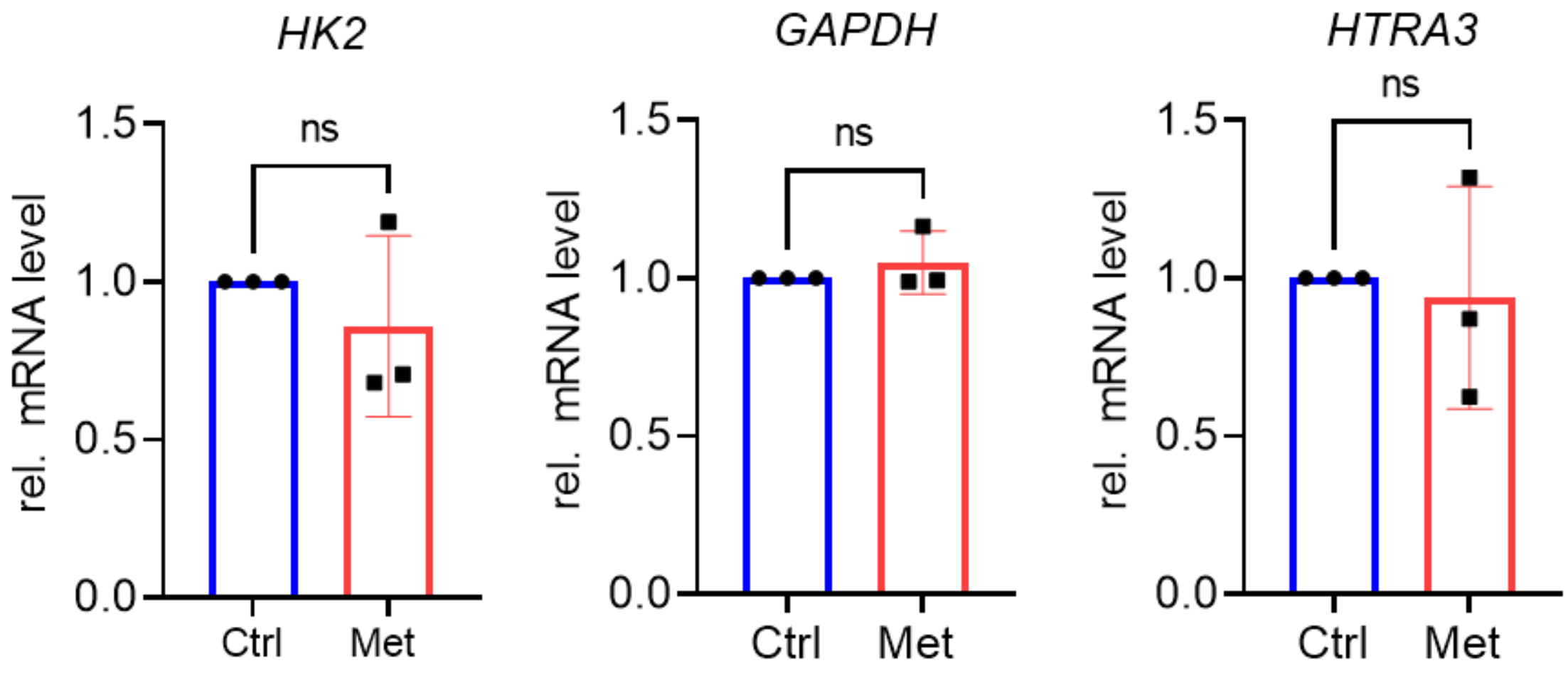

Supplementary Figure 3. Gene expression analysis of selected (pro-invasive) BACH target genes

A549 cells were treated with 10 mM metformin for 24h. After this time, the RNA was isolated and RT-qPCR analyses for *HK2*, *GAPDH* and *HTRA3* are depicted in the graph (n=3, mean ± SD)

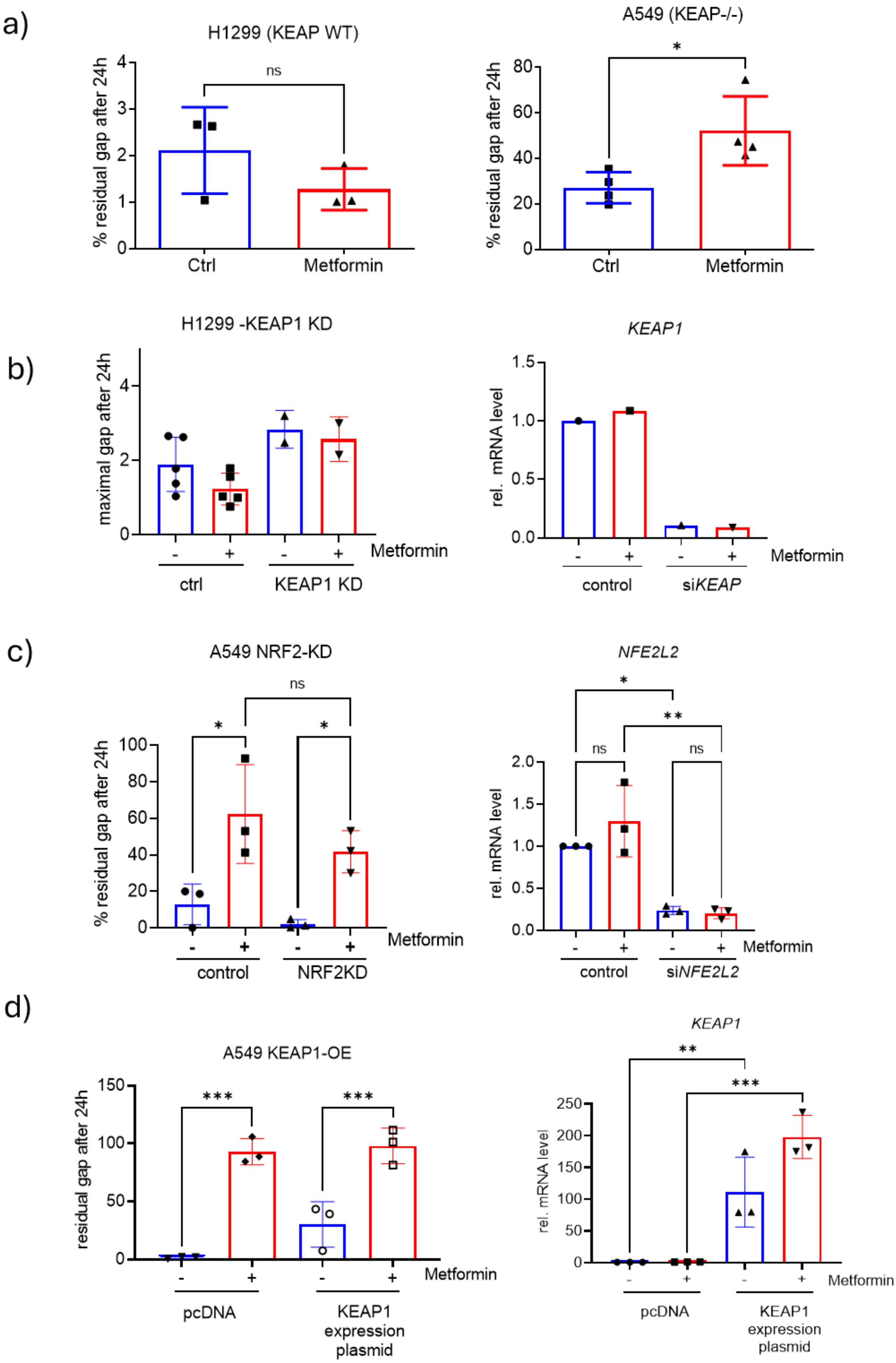
a)
H1299 (KEAP WT)
% residual gap after 24h
ns
Ctrl
Metformin
A549 (KEAP-/-)
% residual gap after 24h
*
Ctrl
Metformin
b)
H1299 -KEAP1 KD
maximal gap after 24h
Metformin
ctrl
KEAP1 KD
KEAP1
rel. mRNA level
Metformin
control
siKEAP
c)
A549 NRF2-KD
% residual gap after 24h
ns
*
*
Metformin
control
NRF2KD
NFE2L2
rel. mRNA level
*
**
ns
ns
Metformin
control
siNFE2L2
d)
A549 KEAP1-OE
residual gap after 24h
***
***
Metformin
pcDNA
KEAP1 expression plasmid
KEAP1
rel. mRNA level
**
***
Metformin
pcDNA
KEAP1 expression plasmid

Supplementary Figure 4. Motility in metformin-treated H1299 *vs* A549 cells upon modulation of NRF2 or KEAP1 expression

(a) Migration (scratch) assays were performed in KEAP WT (H1299) and KEAP-/- (A549) cells with and without 10 mM of metformin. The graphs depict the residual gap after 24h. (n=3, mean ± SD, Mann-Whitney-U-test, *p < 0.05) (b) Scratch assay was performed in H1299 transfected with control siRNA and siRNAs targeting *KEAP1* (left panel). RT-qPCR was carried out to validate the knockdown (right panel). (c) Scratch assay was performed in A549 transfected with a control siRNA and siRNAs targeting *NFE2L2* in the absence and presence of metformin (10 mM) (left panel). Knockdown was cross-checked by RT-qPCR (right panel). (n=3, mean ± SD, Kruskal-Wallis, *p < 0.05, **p<0.01).(d) Scratch assay performed in A549 after overexpression of KEAP1 by an expression plasmid (left panel). Overexpression was cross-checked by RT-qPCR. (n=3, mean ± SD, Kruskal-Wallis, **p < 0.01, ***p<0.001)

Supplementary References

1. Paquette, M., El-Houjeiri, L., C. Zirden, L., Puustinen, P., Blanchette, P., Jeong, H., Dejgaard, K., Siegel, P.M., Pause, A., 2021. AMPK-dependent phosphorylation is required for transcriptional activation of TFEB and TFE3. Autophagy 17, 3957–3975. https://doi.org/10.1080/15548627.2021.1898748

Supplementary Figure 5

Uncropped western blot images.

Please note that some membranes were physically cut to enable simultaneous incubation with different primary antibodies binding to targets of different molecular weight.

For all immunoblots the following molecular weight marker was used

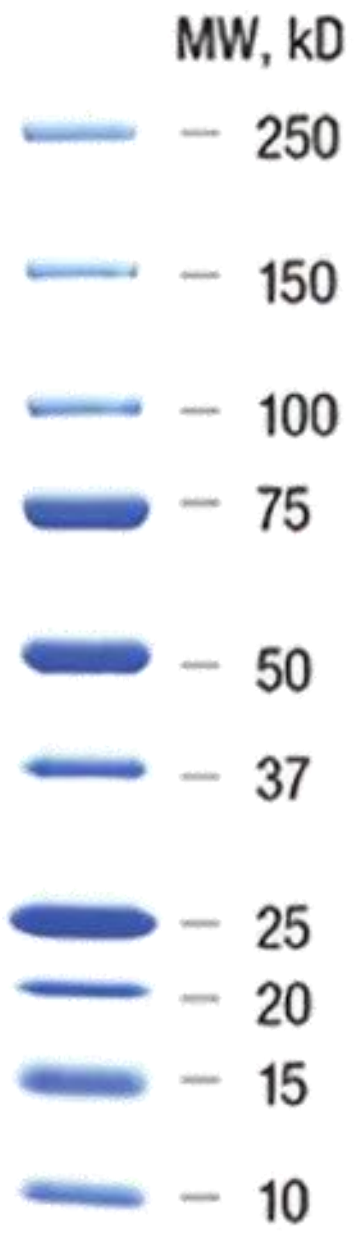

Blots to Figure 1

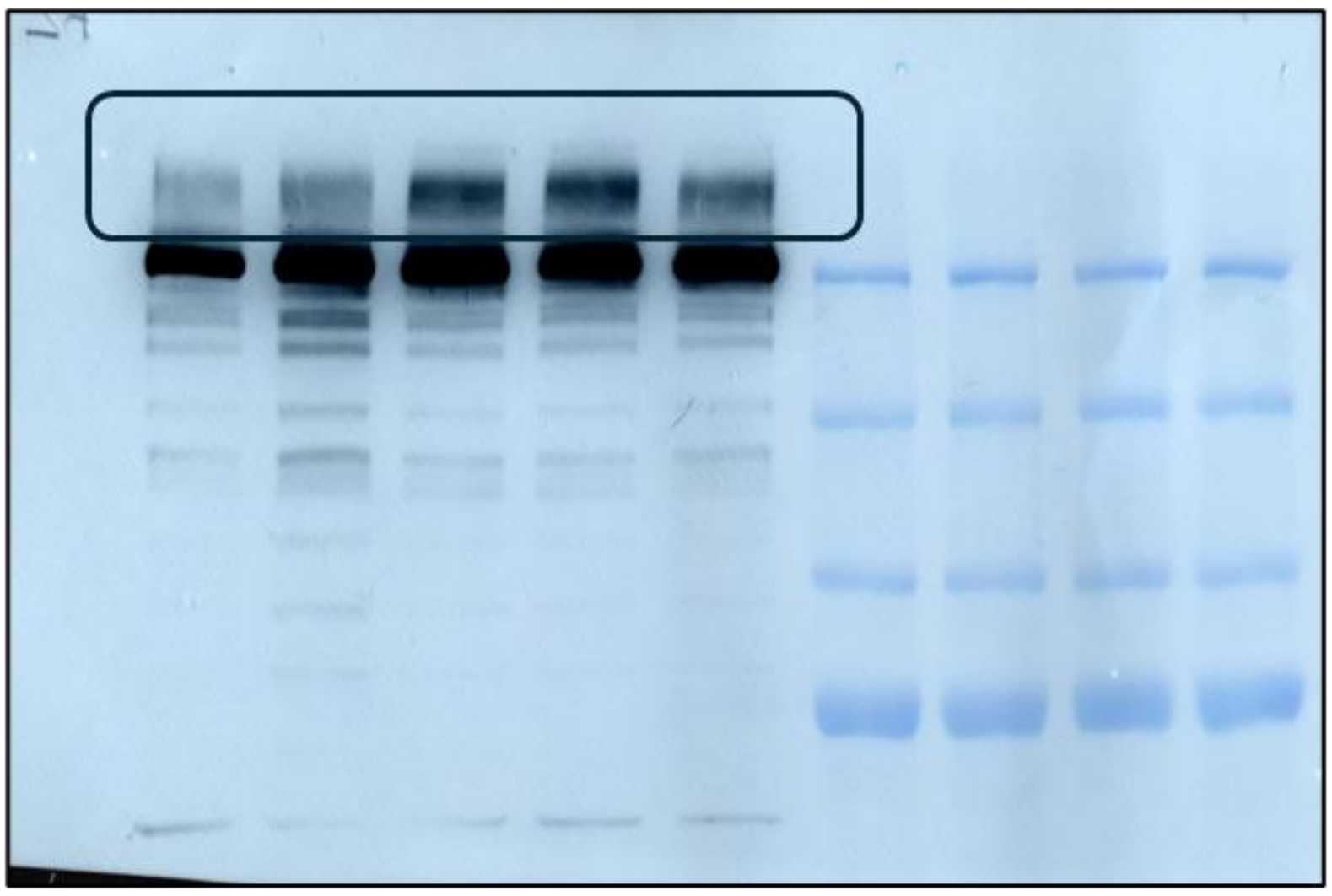

pACC

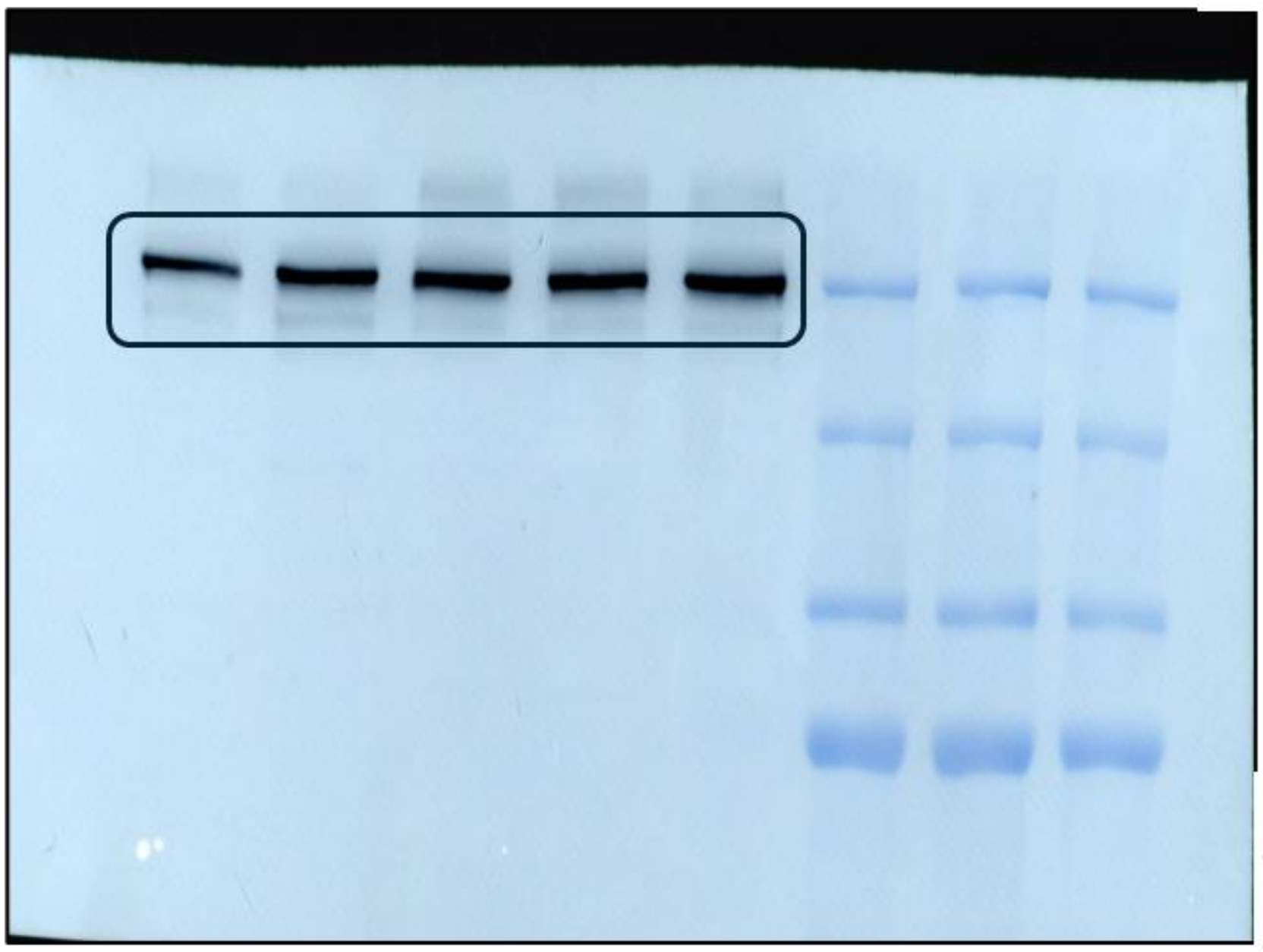

ACC

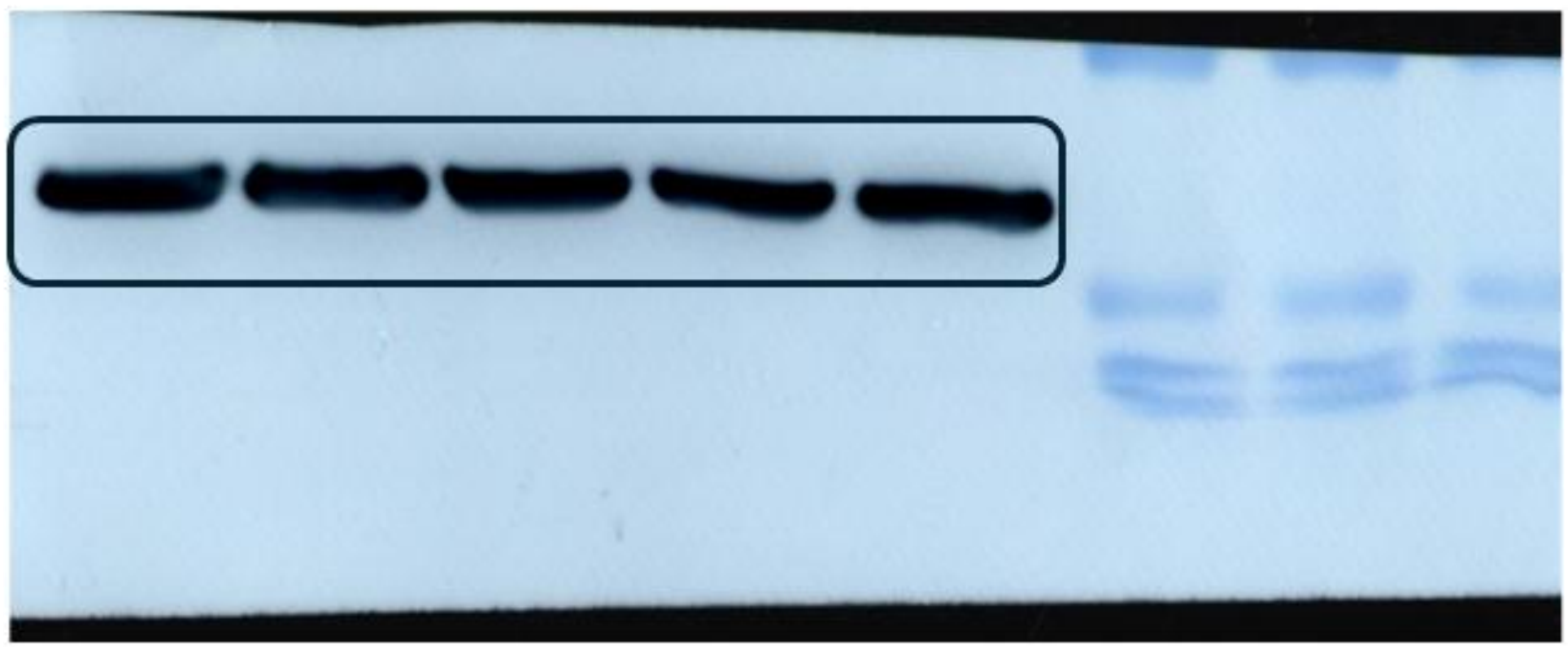

Actin

Blots to Figure 2a)

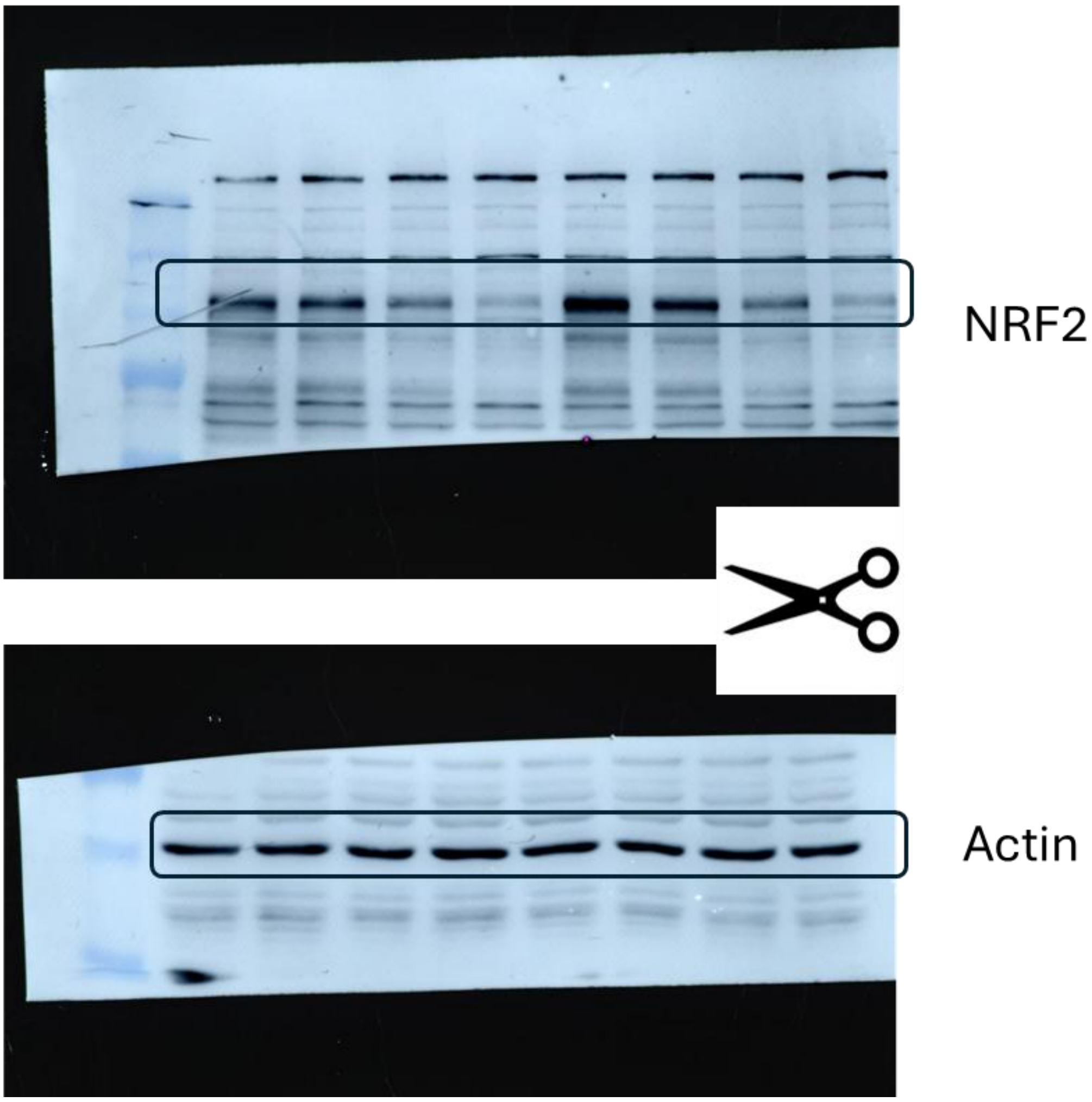

Blots Figure 2c)

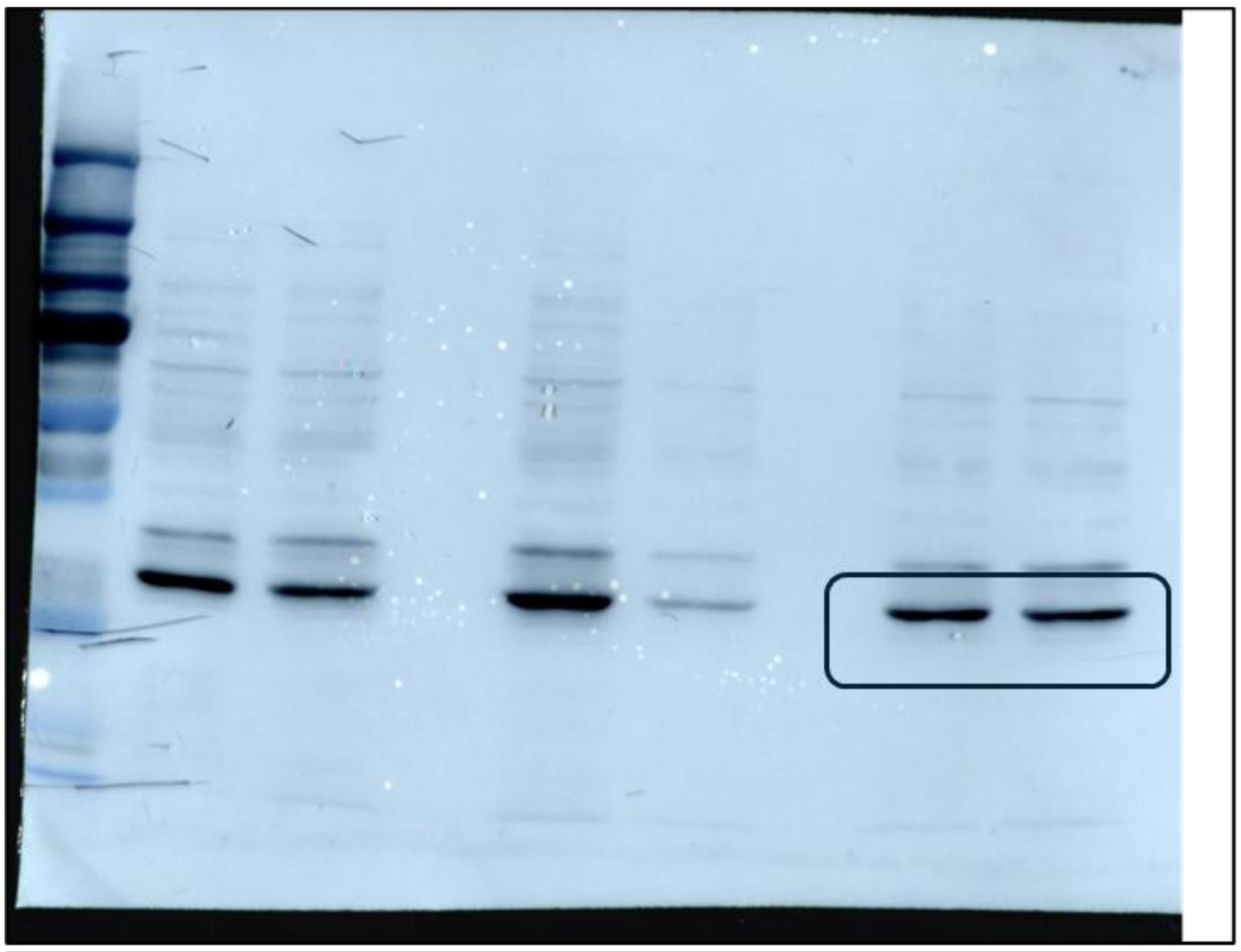

HO1

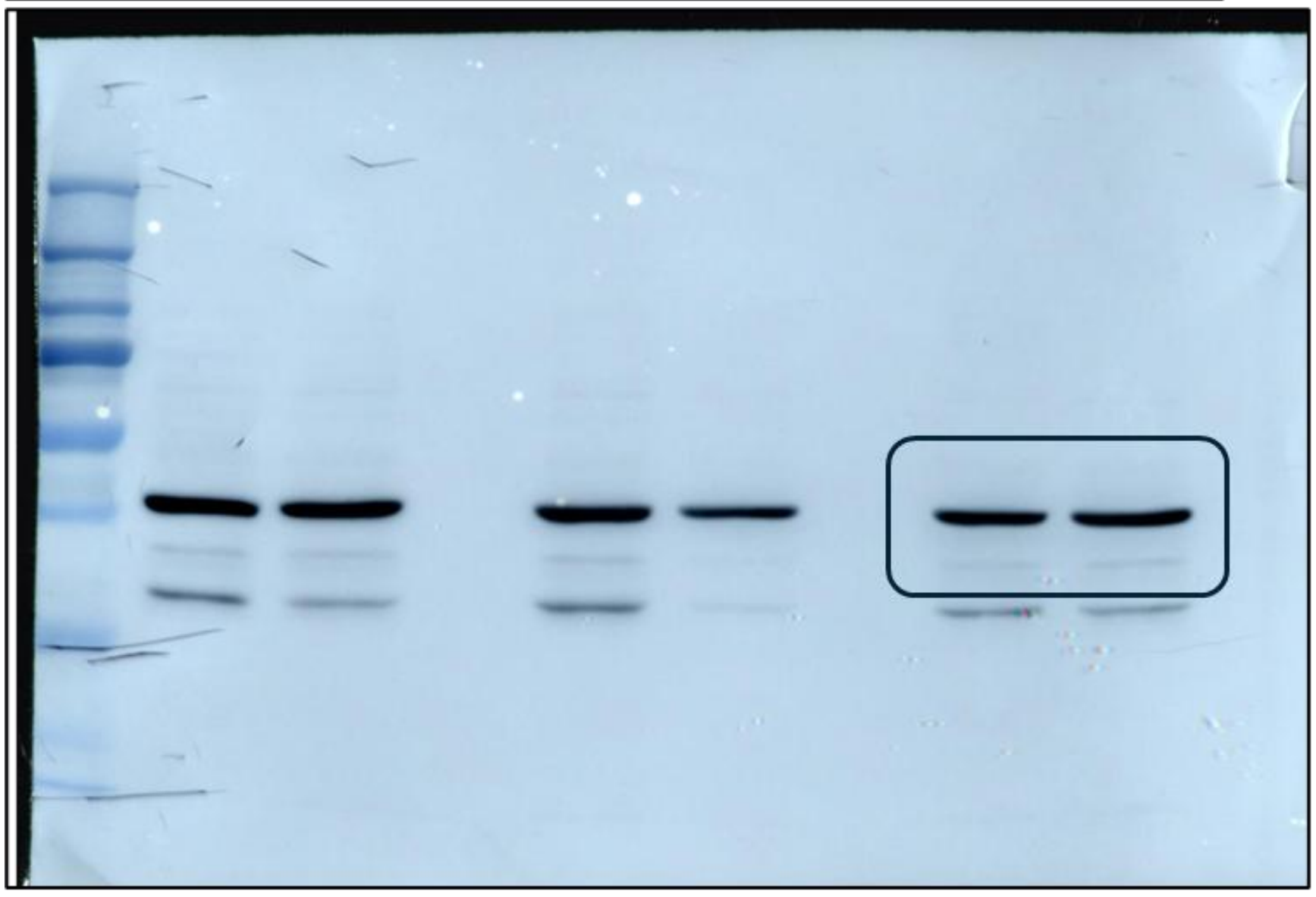

Actin

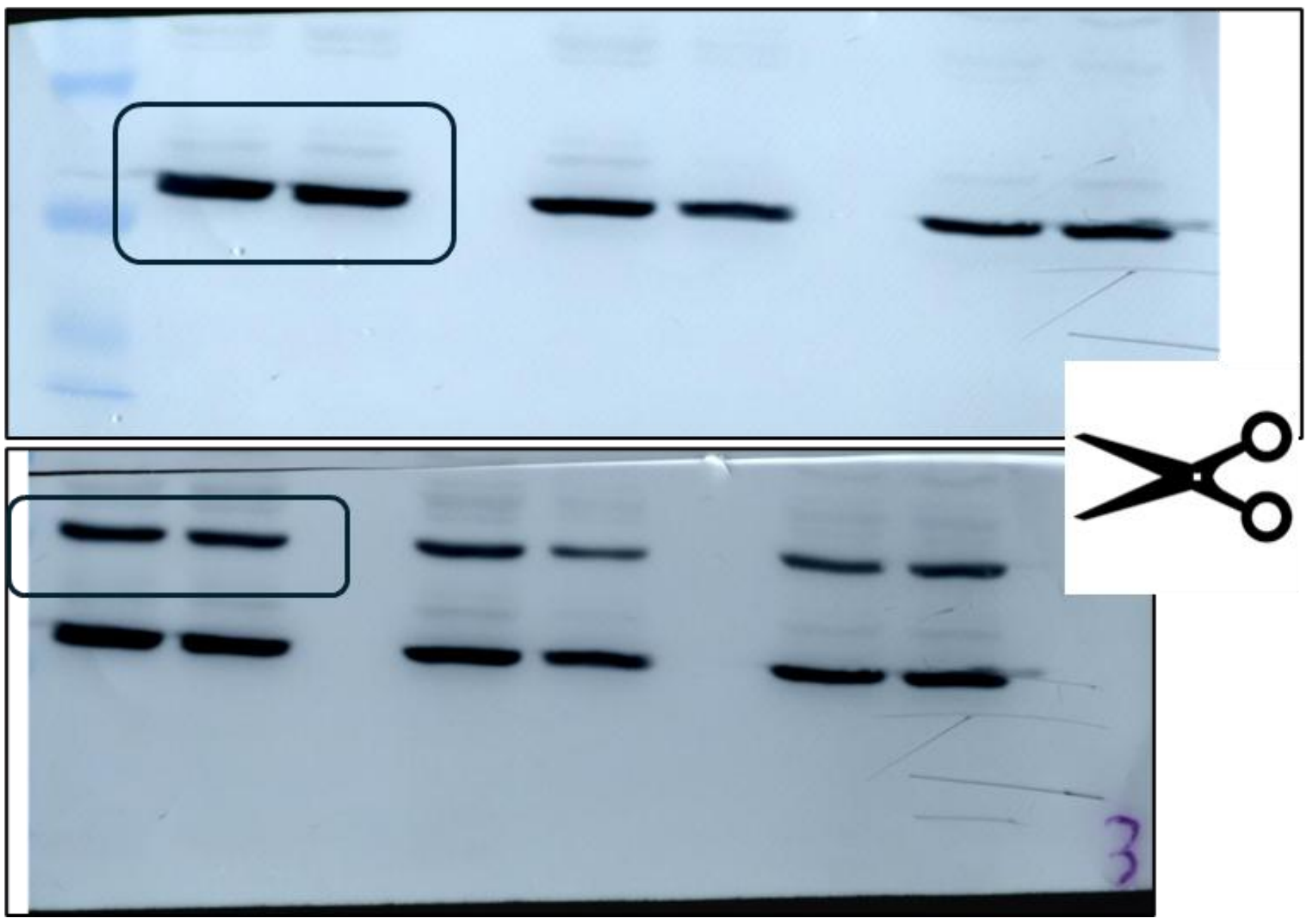

NQO1

ACTIN

Blots Figure 2c) ctd.

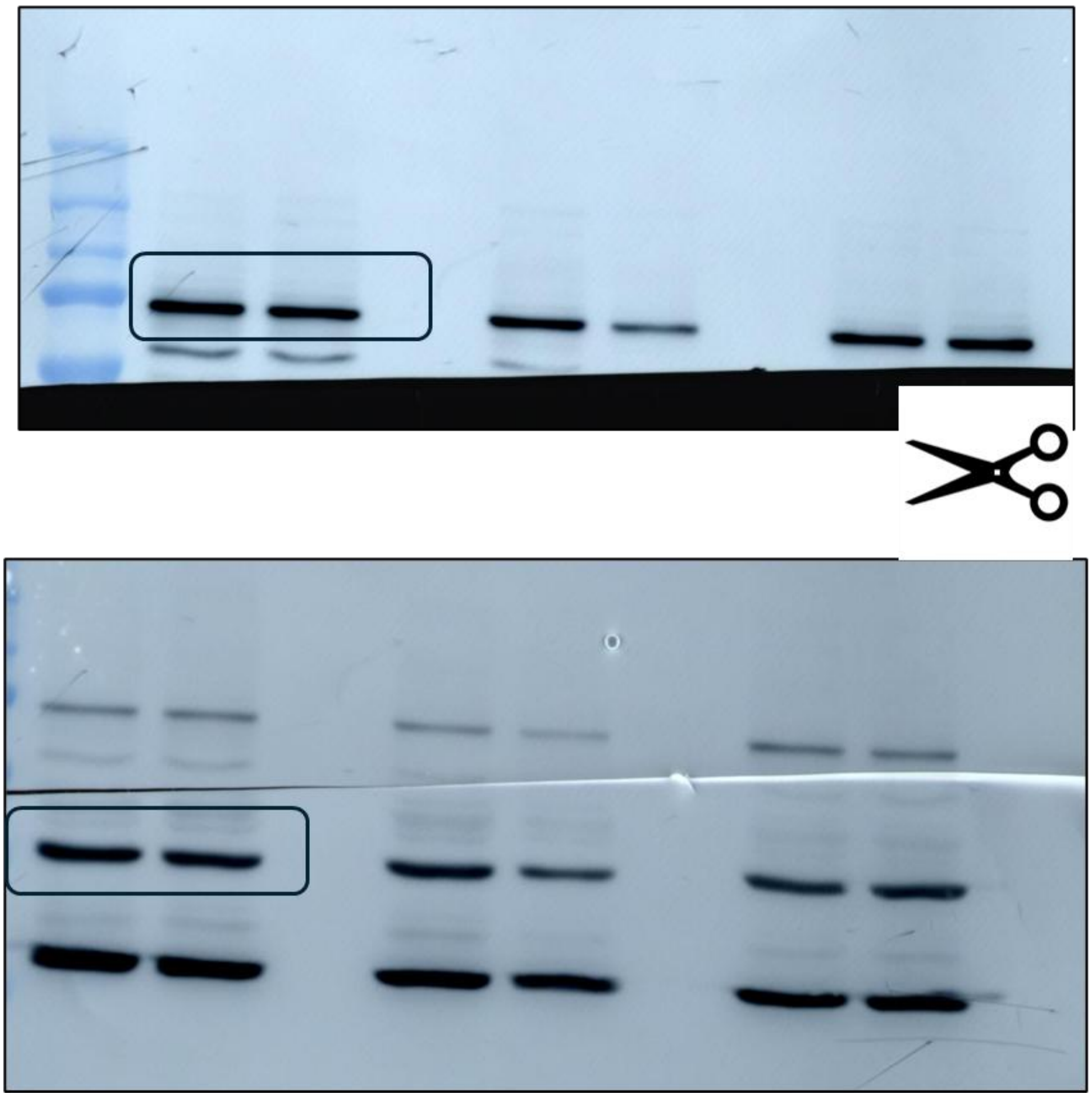

GCLC

Actin

Blots to Figure 3a)

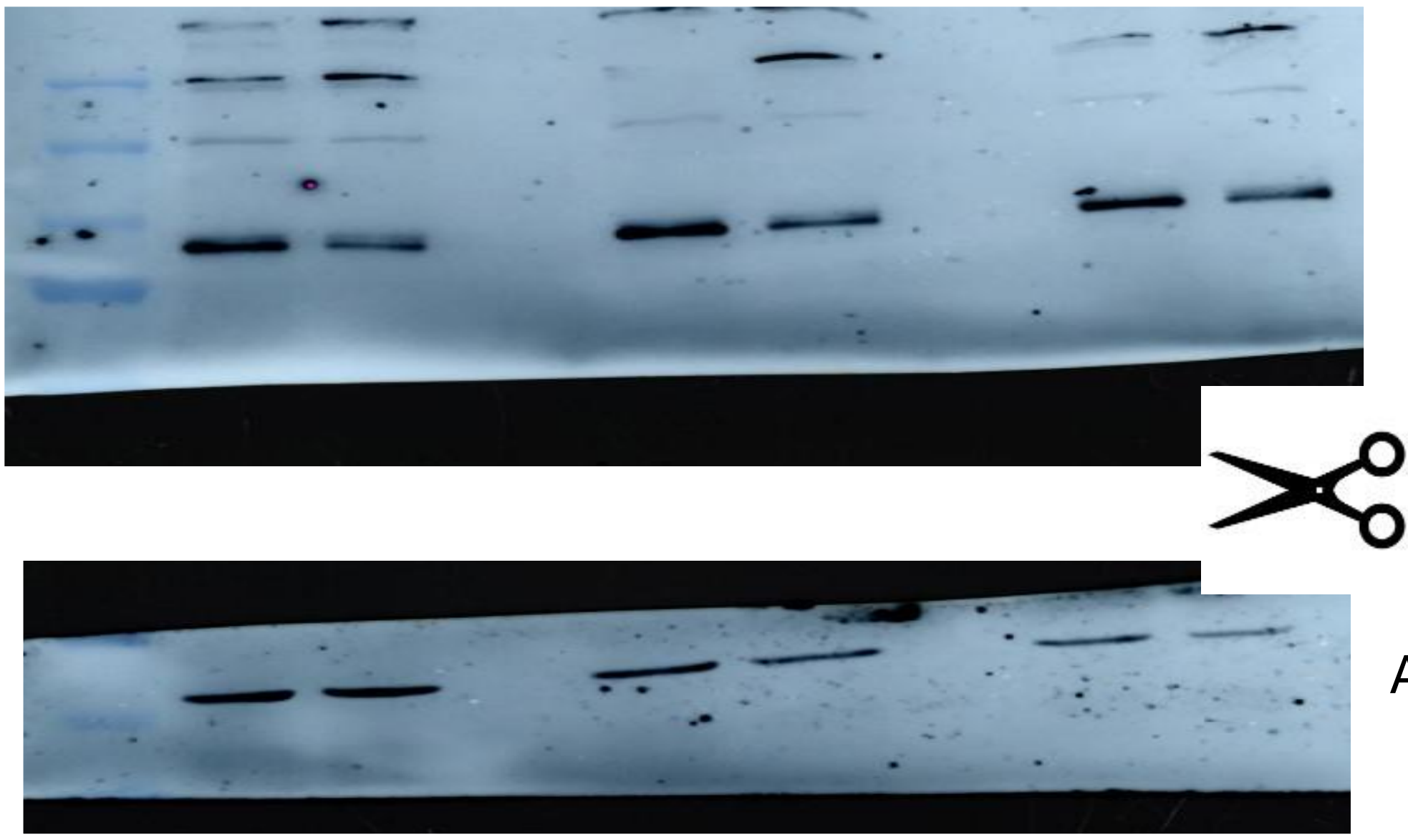

BACH1

Actin

Blots to Figure 5a)

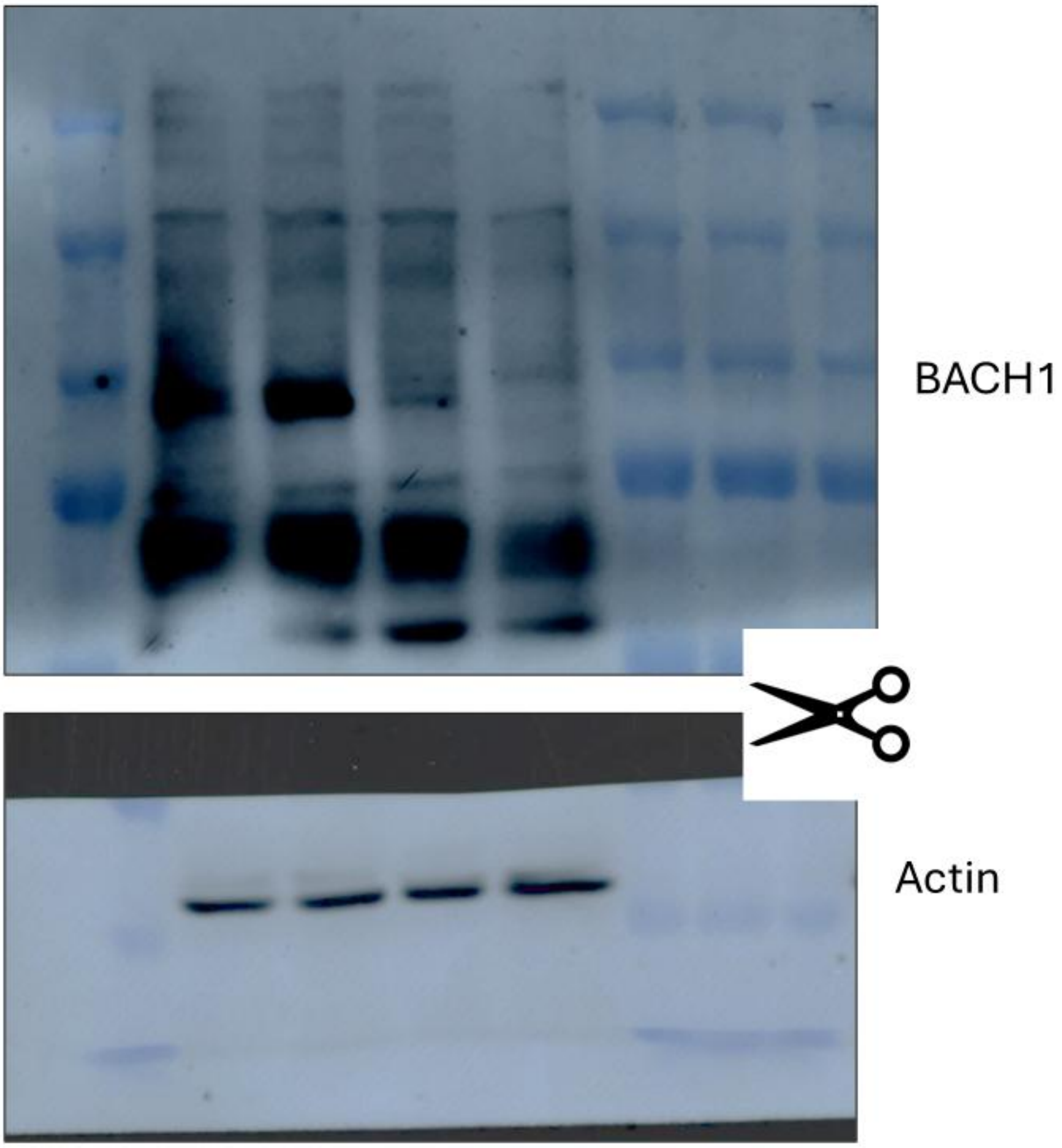

Blots to Figure 5c)

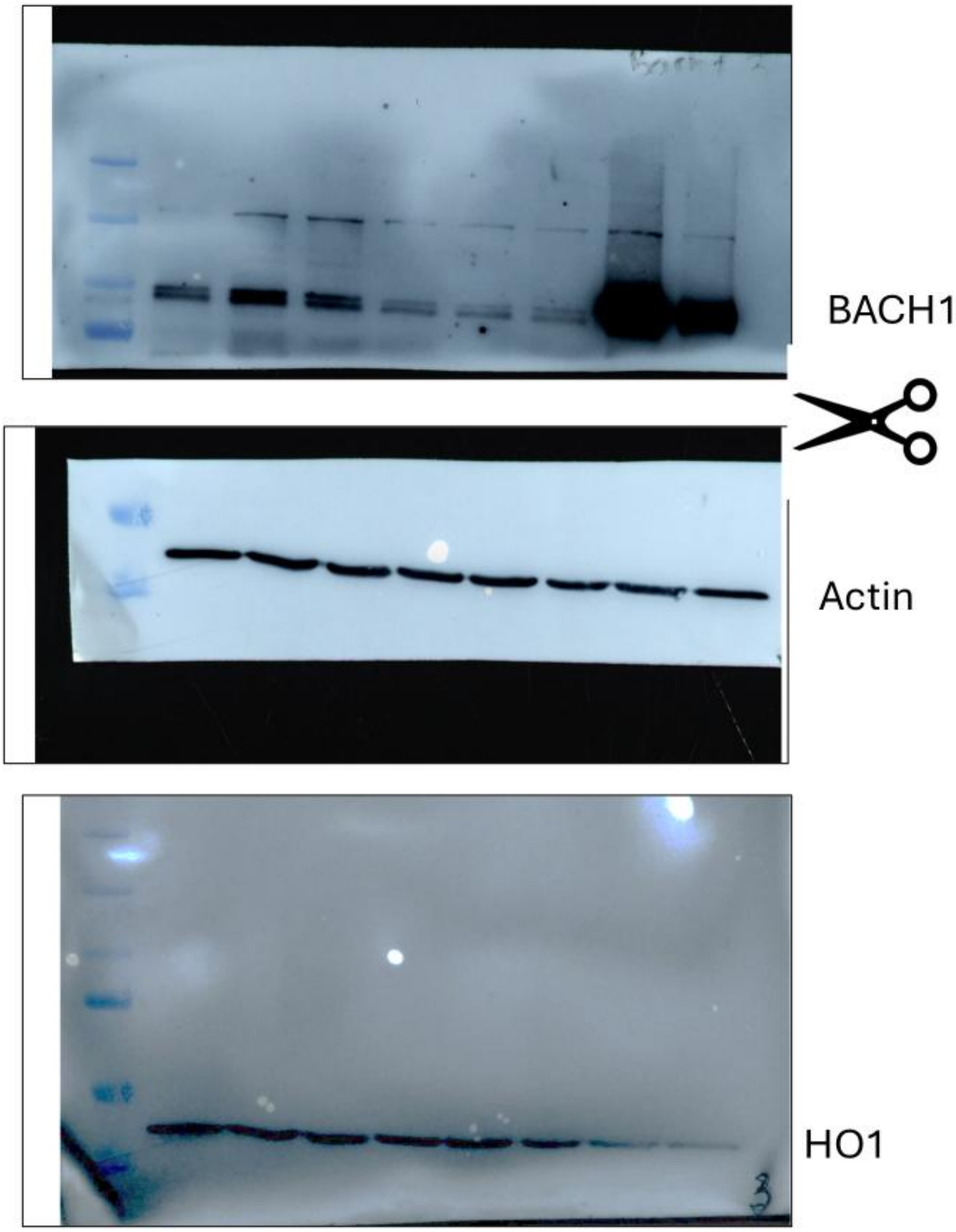

Blots Figure 6b)

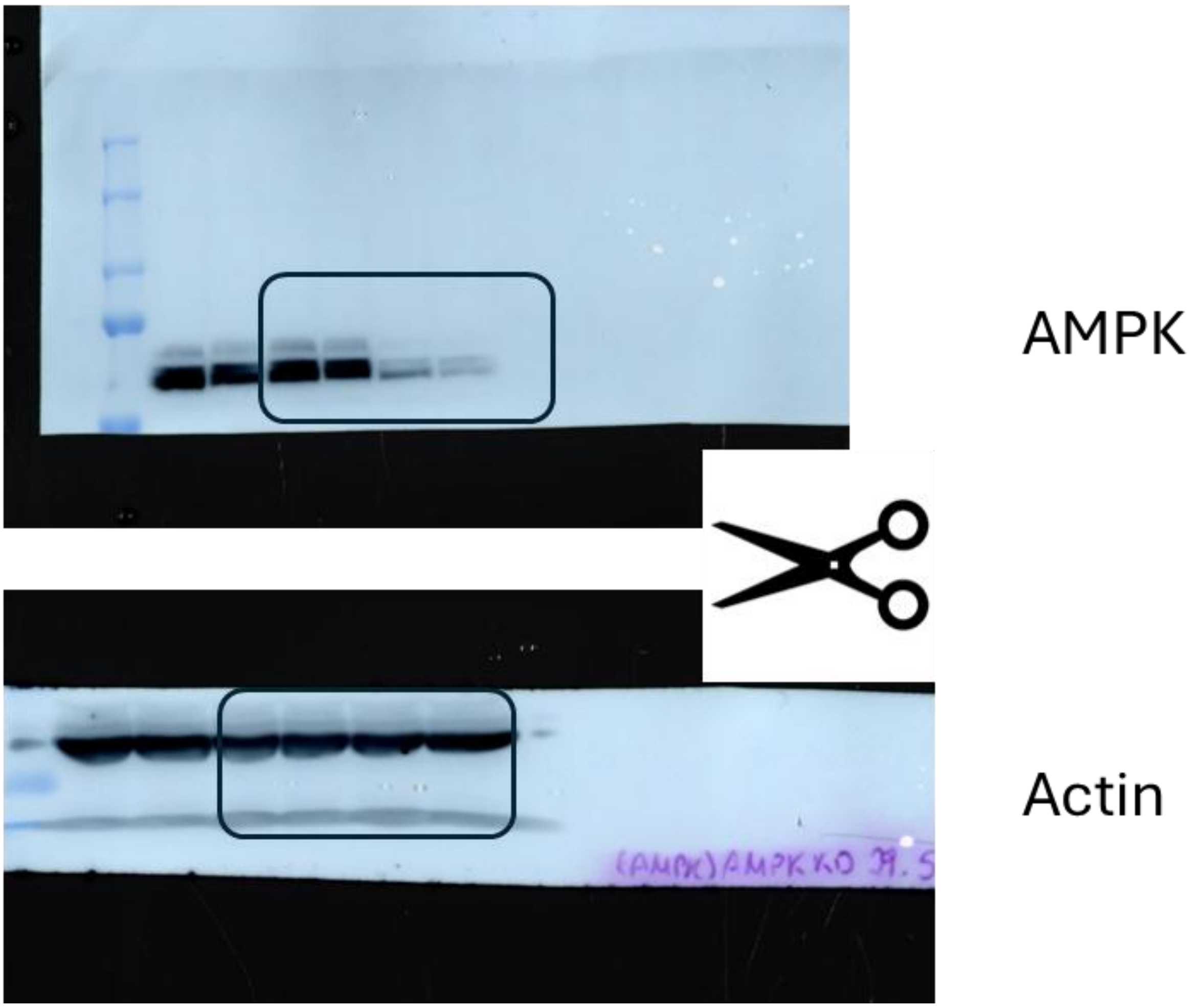

Blots to Figure S1

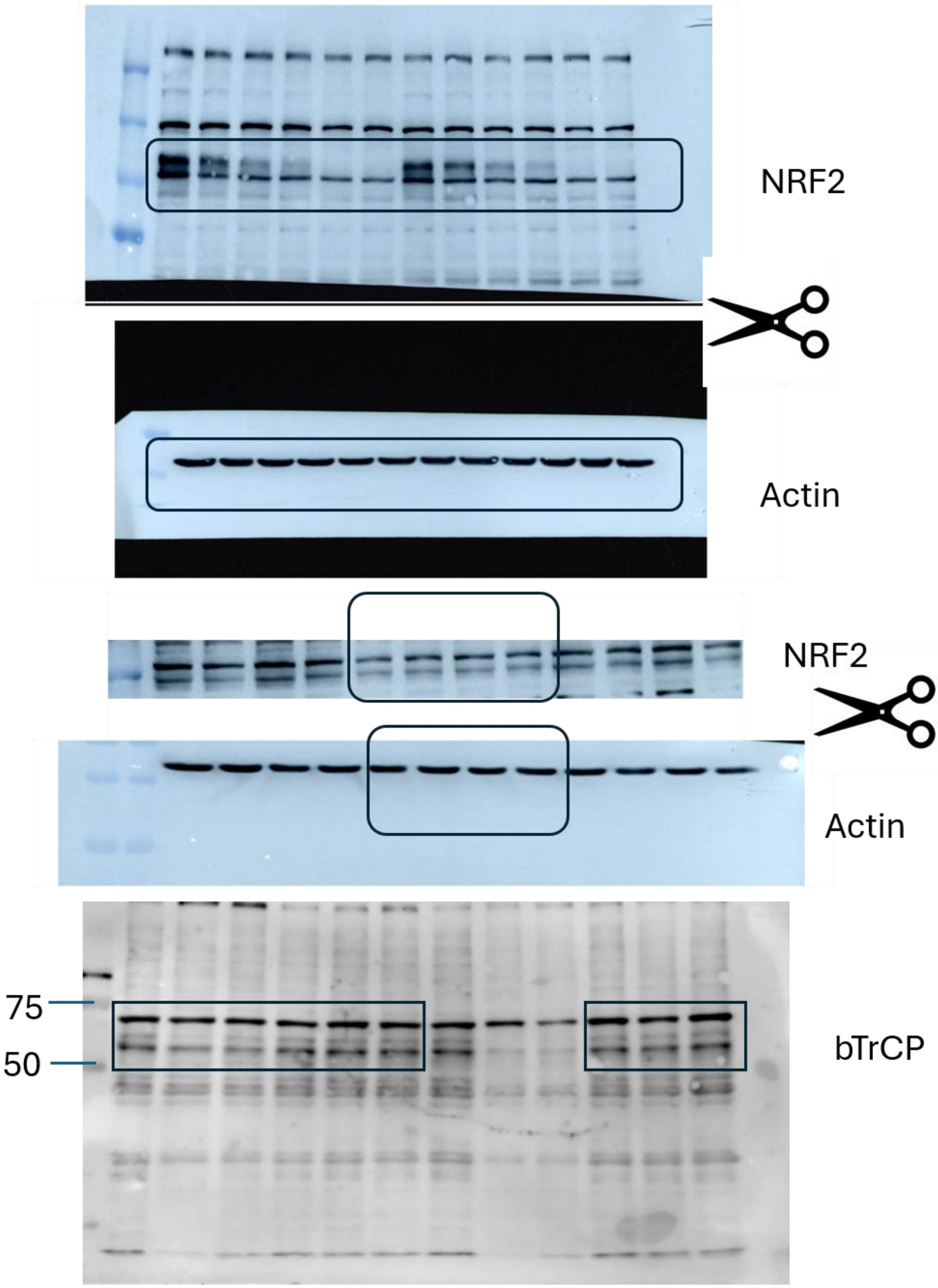

Blots to Figure S1 ctd.

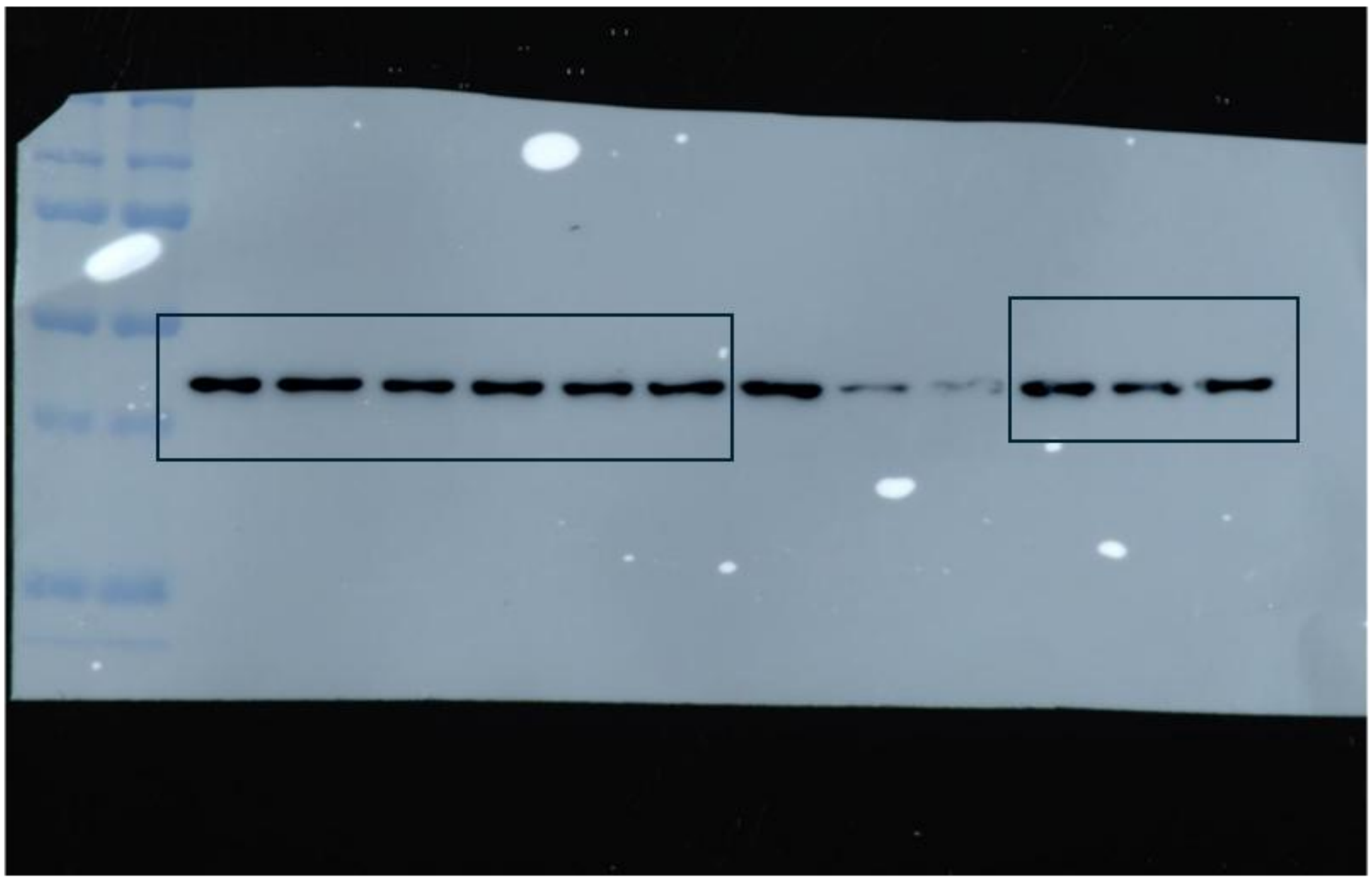

Actin

Blots to Figure S2

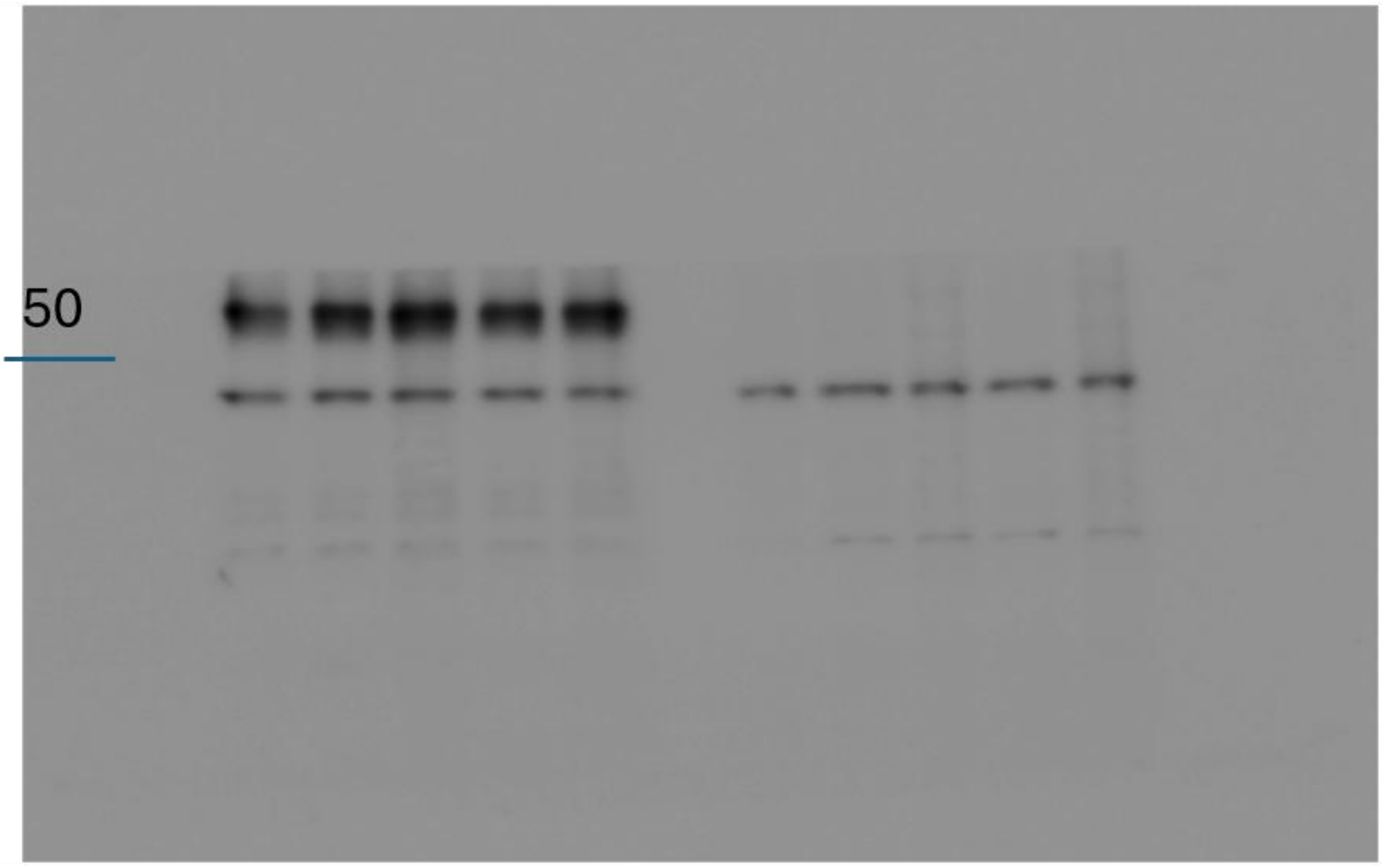

AMPK
